# Multi-step Strategies Toward Regioselectively Sulfated M-Rich Alginates

**DOI:** 10.1021/acs.biomac.3c00045

**Published:** 2023-04-28

**Authors:** Fabiana Esposito, Antonio Laezza, Valentina Gargiulo, Serena Traboni, Alfonso Iadonisi, Annalisa La Gatta, Chiara Schiraldi, Emiliano Bedini

**Affiliations:** † Department of Chemical Sciences, University of Naples Federico II, Complesso Universitario Monte S. Angelo, Via Cintia 4, I-80126 Napoli, Italy; ‡ Department of Sciences, University of Basilicata, Viale dell’Ateneo Lucano 10, I-85100 Potenza, Italy; § Institute of Sciences and Technologies for Sustainable Energy and Mobility, National Research Council (STEMS-CNR), Piazzale V. Tecchio 80, I-80125 Napoli, Italy; ∥ Department of Experimental Medicine, Section of Biotechnology, University of Campania “Luigi Vanvitelli”, Via de Crecchio 7, I-80138 Napoli, Italy

## Abstract

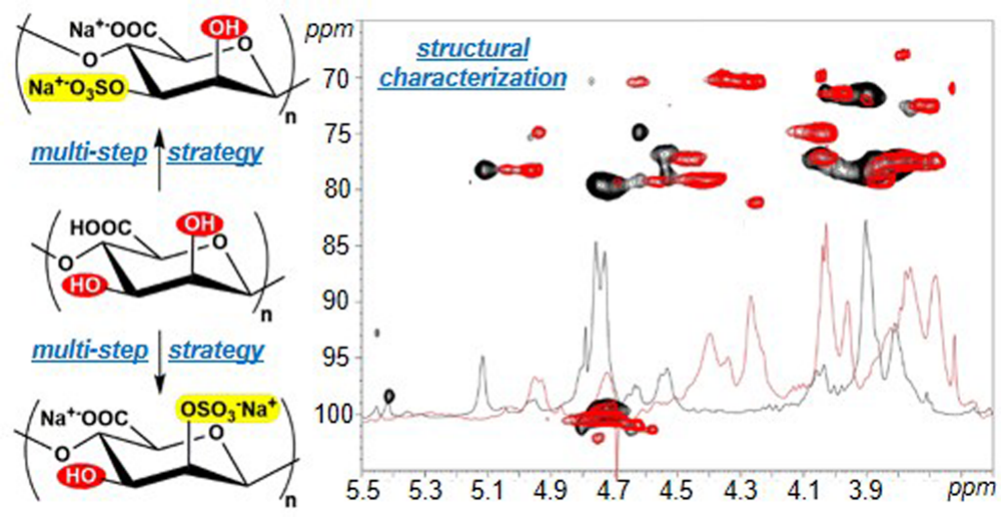

Sulfated alginates (ASs), as well as several artificially sulfated polysaccharides, show interesting bioactivities. The key factors for structure–activity relationships studies are the degree of sulfation and the distribution of the sulfate groups along the polysaccharide backbone (sulfation pattern). The former parameter can often be controlled through stoichiometry, while the latter requires the development of suitable chemical or enzymatic, regioselective methods and is still missing for ASs. In this work, a study on the regioselective installation of several different protecting groups on a d-mannuronic acid enriched (M-rich) alginate is reported in order to develop a semi-synthetic access to regioselectively sulfated AS derivatives. A detailed structural characterization of the obtained ASs revealed that the regioselective sulfation could be achieved complementarily at the O-2 or O-3 positions of M units through multi-step sequences relying upon a silylating or benzoylating reagent for the regioselective protection of M-rich alginic acid, followed by sulfation and deprotection.

## Introduction

**Figure 1:**
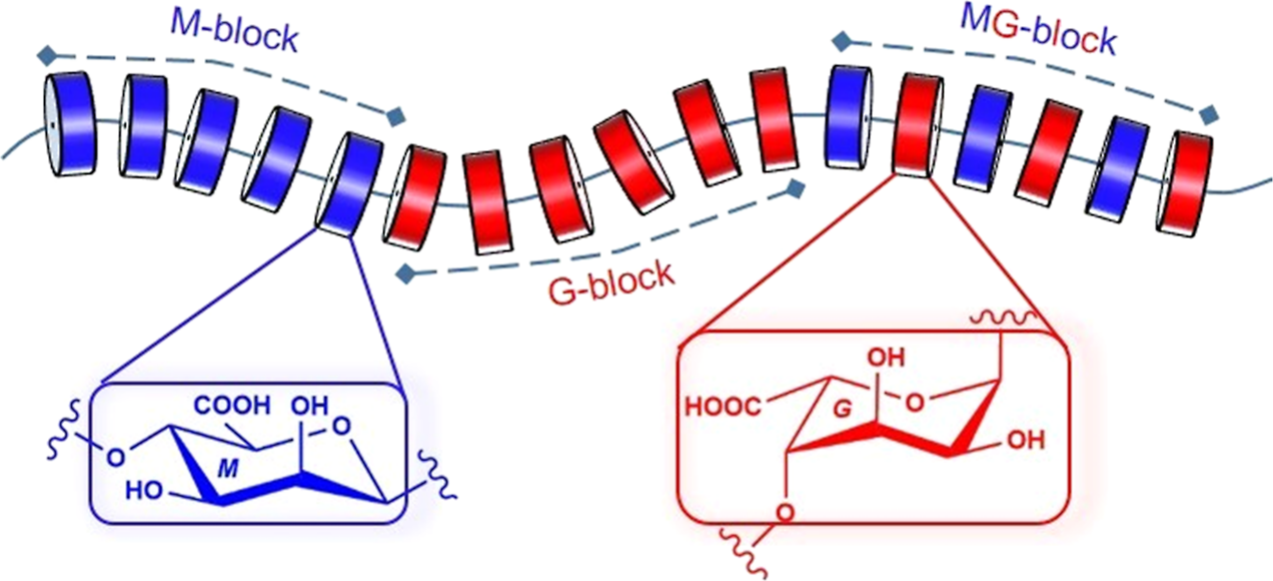
Schematic representation of the chemical structure of alginate polysaccharides.

Alginates are biomacromolecules quite abundant in nature, present in marine brown algae as structural components and in soil bacteria as capsular polysaccharides. They are employed mainly in the food, pharmaceutical, and textile industries for their properties in viscosity enhancement, gel-forming ability, stabilization of aqueous mixtures, dispersions, emulsions, and encapsulation of drugs and cells.^1^ Besides the existing applications for alginates, several others are currently under development.^2,3^ From a structural point of view, alginates are polysaccharides composed of β-1-4-linked d-mannuronic acid (ManA, M) and its C-5 epimer l-guluronic acid (GulA, G) units. These residues are distributed along the polysaccharide chain in a block-copolymer fashion, with homopolymeric regions composed of M-blocks and G-blocks, respectively, and heteropolymeric ones with MG alternating structures (MG-blocks) or more complicated sequences (e.g., GGM- and MMG-)^4^ (Figure 1).

The amount of alginate biosynthesized annually in algae is estimated to be at least ten times higher than the amount produced and employed by industry in the same time period. Such large availability has pushed chemists to investigate the possibility of obtaining structurally modified alginate polysaccharides as novel, sustainable materials with enhanced properties with respect to the unmodified polysaccharide or completely new activities otherwise not existing in the alginate’s native form. Several kinds of transformations have been reported.^5−7^ Among them, one of the most investigated is the insertion of sulfate groups on the alginate backbone.^8^ Sulfated alginates (ASs), as well as several other artificially sulfated polysaccharides,^9−12^ show interesting anti-coagulant, anti-inflammatory, and wound dressing activities, often associated with unique features and properties with respect to glycosaminoglycans (GAGs), which are the natural sulfated polysaccharides employed as drugs for the treatment of several pathologies. Furthermore, ASs and most artificially sulfated polysaccharides can be produced from renewable, largely available raw materials, thus standing as a more environmentally and economically sustainable alternative to sulfated GAGs, which are commonly extracted from animal tissues. Last but not least, they do not present some additional problems raised by sulfated GAGs use in medicine,^13^ such as the risk of contamination with animal proteins, the difficulty in their isolation as single GAGs in pure form and in controlling the degree of sulfation (DS), and the distribution of sulfate groups along the polysaccharide backbone (sulfation pattern). Indeed, these structural features depend not only on animal species and tissue but also on the physiopathological conditions, e.g., aging, inflammation, and tumor formation, of the individual animal.^14,15^

Notably, a low-molecular-weight alginate sulfated derivative (propylene glycol alginate sodium sulfate, PSS) has been used for more than 30 years in China as a heparin-like drug for the treatment of cardiovascular diseases. Nonetheless, a remarkable number of adverse events have been recorded during the clinical employment of PSS.^16^ The M/G ratio is known to play an important role in both anticoagulant and side effects of PSS,^17^ but the key factors for structure–activity relationship (SAR) studies, not only for PSS but also for every sulfated polysaccharide, are the DS and, above all, the sulfation pattern.^18^ The latter is indeed known to encode specific functional information in natural polysaccharides, such as sulfated GAGs, and many efforts are devoted to unveiling the role played by such sulfation codes in a huge number of biological events.^19^ In artificially sulfated polysaccharides, DS can often be regulated through stoichiometry, while the control of sulfation pattern requires the development of chemical or enzymatic methods for regioselective insertion of sulfate groups exclusively at determined positions. Although this goal has been achieved for several polysaccharides up to now, to the best of our knowledge, it is still missing for any AS derivative.^8,20^ Indeed, quite good control of DS on alginates with homogeneous monosaccharide sequences (poly-M, poly-G, and poly-MG) could be achieved,^21^ whereas no control of sulfation pattern—neither between nor within M and G units (i.e., M- vs G- and O-2 vs O-3, respectively)—has been reported. Only a slight preference for sulfation of M- over G-units and of O-3 over O-2 positions has been observed, anyway, in the context of an undoubtedly random sulfation pattern.^22^

Methods commonly employed for regioselective installation of sulfate groups on a polysaccharide are based on direct, regioselective sulfation reactions or multi-step procedures consisting of regioselective protection(s)-sulfation-deprotection sequences.^20^ The former approach has been already attempted in the case of AS semi-synthesis, unfortunately with no success.^8^ Conversely, to the best of our knowledge, the latter strategy has not been reported yet for ASs. Therefore, in this work, we have screened several different protecting groups that are known to allow regioselective protections on mono- and/or oligosaccharides to evaluate whether regioselectively sulfated derivatives of a commercially available M-rich alginic acid could be accessible by suitable multi-step, semi-synthetic sequences or not. In particular, both 2-O- and 3-O-selectively protected polysaccharide derivatives have been pursued in order to obtain for the first time AS products differently sulfated at 3-O- or 2-O-positions (M3S or M2S), respectively. A detailed structural characterization of the semi-synthesized ASs was then performed, showing that in some cases regioselective sulfation was efficiently gained.

## Experimental Section

### General Methods

Commercial-grade reagents and solvents were used without further purification, except where indicated otherwise. M-rich alginic acid (molecular weight = 9 ± 2 kDa, polydispersity index = 1.3 ± 0.3) was purchased from Biosynth (Thal, Switzerland). Its M/G composition was evaluated by ^1^H NMR anomeric peak integration (see Figure S15 in Supporting Information), in agreement with the literature:^23^ M = 83%, G = 17%; MM = 57%, MG = GM = 17%, GG = 1%; reducing M = 8%). The term “pure water” refers to water purified by a Millipore Milli-Q Gradient system (Merck Millipore, Burlington, MA, USA). Dialyses were conducted on Spectra/Por 3.5 kDa cut-off membranes at 4 °C. Centrifugations were performed with an Eppendorf (Hamburg, Germany) Centrifuge 5804R instrument at 4 °C (4600*g*, 5 min). Freeze-drying was performed with a 5Pascal (Trezzano sul Naviglio, Italy) Lio 5P 4K freeze dryer. Elemental analyses were performed on Leco 628 and Leco 144 Elemental Analyzer instruments (Leco, St. Joseph, MI, USA). NMR spectra were recorded on a Bruker (Billerica, MA, USA) Avance-III HD (^1^H: 400 MHz, ^13^C: 100 MHz) or on a Bruker Avance-III (^1^H: 600 MHz, ^13^C: 150 MHz) instrument—the latter equipped with a cryo-probe—in D_2_O (acetone as the internal standard, ^1^H: (CH_3_)_2_CO at δ 2.22 ppm; ^13^C: (CH_3_)_2_CO at δ 31.5 ppm) or DMSO-*d*_6_ (^1^H: CHD_2_SOCD_3_ at δ 2.49 ppm; ^13^C: CD_3_SOCD_3_ at δ 39.5 ppm). The data were processed using the data analysis packages integrated with Bruker TopSpin 4.0.5 software. Gradient-selected COSY and NOESY experiments were performed using spectral widths of 6000 Hz in both dimensions, and data sets of 2048 × 256 points. ^1^H,^13^C-HSQC and ^1^H,^13^C-HMBC experiments were measured in the ^1^H-detected mode via single quantum coherence with proton decoupling in the ^13^C domain, using data sets of 2048 × 256 points and typically 80 increments (160 for HMBC). A Viscotek TDA 305 instrument (Malvern, UK) was used to determine molecular mass data.

### Typical Procedure for Silyl Ether Installation

Derivative **1** (44.7 mg) was suspended in dry *N*,*N*-dimethylformamide (DMF, 1.7 mL) and then treated with *t*-butyl-dimethylsilyl chloride (TBDMSCl, 177 mg) and imidazole (240 mg). The mixture was stirred overnight at 50 °C. Thereafter, it was cooled to rt and treated with diisopropyl ether (7 mL). The mixture was stored at −28 °C for some hours; then, the obtained precipitate was collected by centrifugation and dried under vacuum to give derivative **2** (120 mg, 268% weight yield).

### Typical One-Pot Procedure for Orthoester Installation and Cleavage

M-rich alginic acid (85.0 mg, 0.481 mmol repeating unit) was suspended in dry DMF (3.8 mL). After 90 min stirring at rt, trimethyl orthobenzoate (PhC(OCH_3_)_3_, 752 μL, 4.38 mmol) and then (+)-camphor-10-sulfonic acid (CSA, 140 mg, 603 μmol) were added. The suspension was stirred at rt overnight; then, pure water (15 mL) was added. The resulting milky suspension was stirred for 2 h, then subjected to dialysis and freeze-drying. The whole procedure was performed twice. Polysaccharide **5-iii** (94.4 mg, 111% weight yield) was obtained as a yellowish powder.

### Typical Procedure for Benzylidene Installation

A suspension of M-rich alginic acid (196 mg, 1.11 mmol repeating unit) in dry DMF (15 mL) was briefly stirred at 80 °C and then treated at rt with α,α-dimethoxytoluene (PhCH(OCH_3_)_2_, 816 μL, 5.44 mmol) that was freshly dried over 4 Å molecular sieves and then with a 0.21 M solution of CSA in dry DMF (0.77 μL). The mixture was vigorously stirred at 80 °C overnight, then cooled to rt and treated with diisopropyl ether (40 mL) to give a white precipitate. The mixture was stored at −28 °C for some hours, and solids were collected by centrifugation. After overnight drying under vacuum, the derivative **7-i** (175 mg, 89% weight yield) was obtained as a white powder.

### Typical Procedures for Sulfation and Global Deprotection

Polysaccharide **5-v** (180 mg) was suspended in dry *N*,*N*-dimethylformamide (DMF, 5.2 mL) and then treated with a 1.45 M solution of pyridine–sulfur trioxide complex (SO_3_·py) in dry DMF (7.5 mL). After overnight stirring at 50 °C, a saturated NaCl solution in acetone (11 mL) was added at rt, and the mixture was then stored at −28 °C for some hours. The obtained white precipitate was collected by centrifugation and then dissolved in pure water (5.0 mL), and the resulting acid mixture (pH ∼ 2) was heated to 50 °C. After 2 h of stirring at 50 °C, the mixture was cooled and then treated with a 4 M NaOH_(aq)_ solution to adjust pH to 12. The solution was stirred at rt overnight and then neutralized with 1 M HCl_(aq)_. Dialysis and subsequent freeze-drying gave polysaccharide **AS-7** (266 mg, 148% weight yield) as a white amorphous solid.

### Methyl Esterification of Starting M-Rich Alginic Acid

M-rich alginic acid (520 mg, 2.95 mmol repeating unit) was treated via cannula with a solution obtained by dissolving concentrated H_2_SO_4_ (535 μL) in dry methanol (103 mL) under an Ar atmosphere. The mixture was stirred at 60 °C for 24 h; then, the solid was collected by centrifugation, followed by washing with 1:1 v/v EtOH–acetone. After drying the solid under vacuum, derivative **1** (292 mg, 56% weight yield) was obtained.

### Benzoylation of M-Rich Alginate Methyl Ester **1** with Bz_2_O

Derivative **1** (61.4 mg) was dissolved in dry DMF (2.3 mL) and then treated with benzoic anhydride (Bz_2_O, 362 mg) and *N*,*N*-diisopropylethylamine (DIPEA, 112 μL). The solution was stirred overnight at rt and then treated with diisopropyl ether (11 mL). The mixture was stored at −28 °C for some hours; then, the obtained precipitate was collected by centrifugation and dried under a vacuum to give product **5-i** (91.3 mg, 149% weight yield).

### Benzoylation of M-Rich Alginate Methyl Ester **1** with BzCN

Derivative **1** (59.8 mg) was dissolved in 9:1 v/v DMF-pyridine (2.5 mL) and then treated with benzoyl cyanide (BzCN, 206 mg) and 4-(dimethylamino)pyridine (DMAP, 10.6 mg). The solution was stirred overnight at rt and then treated with methanol (2 mL) and diisopropyl ether (10 mL). The mixture was stored at −28 °C for some hours; then, the obtained precipitate was collected by centrifugation and dried under a vacuum to give product **5-ii** (121 mg, 202% weight yield).

### Oxidative Cleavage of Benzylidene CPGs on M-Rich Alginic Acid Derivative **7-i**

Polysaccharide **7-i** (473 mg) was suspended in ethyl acetate (20 mL) and treated with a 0.27 M solution of sodium bromate in pure water (20 mL) and then with a 0.24 M solution of sodium dithionite in pure water (18 mL). The addition of the latter solution was carried out in five aliquots at a few minute intervals. The triphasic reaction mixture was vigorously stirred at rt overnight. Thereafter, the yellowish solid was collected by centrifugation and dried under vacuum overnight. The biphasic liquid mixture was partitioned in a separation funnel, the water phase collected, and then subjected to dialysis and freeze-drying. The obtained yellowish residue was combined with the previously collected solid to afford the derivative **5-v** (190 mg, 40% weight yield).

### Oxidative Cleavage of Benzylidene CPGs on M-Rich Alginate Methyl Ester Derivative **7-ii**

Polysaccharide **7-ii** (52.2 mg) was suspended in ethyl acetate (1.3 mL) and treated with a 0.27 M solution of sodium bromate in pure water (1.3 mL) and then with a 0.24 M solution of sodium dithionite in pure water (1.2 mL). The addition of the latter solution was carried out in five aliquots at a few minute intervals. The biphasic reaction mixture was vigorously stirred at rt overnight. Thereafter, the biphasic liquid mixture was partitioned by centrifugation, the organic phase collected, dried over anhydrous Na_2_SO_4_, filtered, and concentrated by rotary evaporation to give the derivative **5-vi** (34.5 mg, 66% weight yield) as a yellowish residue.

### One-Pot Lactonization-Benzoylation-Lactone Opening Sequence on M-Rich Alginic Acid

M-rich alginic acid (196 mg, 1.11 mmol repeating unit) was suspended in dry DMF (8.8 mL) and briefly stirred at 85 °C. The resulting, fine, yellowish suspension was treated with Bz_2_O (7.57 g, 33.5 mmol) at rt under an argon atmosphere. The mixture was vigorously stirred at 85 °C for 26 h, then cooled to rt and treated with pyridine (4.9 mL) and DMAP (182 mg, 1.49 mmol). After 72 h further stirring at rt, dry methanol (7.5 mL) and sodium acetate (NaOAc, 137 mg, 1.67 mmol) were added, and stirring was continued for 24 h. Thereafter, a further amount of methanol (16 mL) was added, and the reaction mixture was concentrated to approximately 20 mL volume by rotary evaporation. The residue was treated at 0 °C with diisopropyl ether (9 mL) to give a white precipitate. The mixture was stored at −28 °C for some hours; then, the solid was collected by centrifugation and further purified by dissolving it in DMSO (9 mL) and then precipitating with 2:1 v/v acetone-diisopropyl ether (45 mL). After overnight drying under vacuum, derivative **5-vii** (190 mg, 97% weight yield) was obtained as a white powder.

### Degree of Sulfation Evaluation by Elemental Analysis

C, H, and N contents were determined in accordance with the ASTM D3176 protocol by using a Leco 628 elemental analyzer (Leco, St. Joseph, MI, USA). Ethylenediaminetetraacetic acid (EDTA) was used as reference material for instrument calibration, and each measurement was repeated twice. The S content was measured in accordance with the ASTM D4239 protocol by using a Leco 144 analyzer. Vanadyl sulfate pentahydrate and a low sulfur coal (Leco 502-681) were used as reference materials for instrument calibration, and each measurement was repeated twice. DS values were calculated according to eq 1 (*m*_C_ = atomic mass carbon, *m*_S_ = atomic mass sulfur, and *n*_C_ = number of carbon atoms per repeating unit).
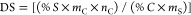
1

### Hydrodynamic Analyses

A detailed hydrodynamic analysis was carried out as previously described.^24^ Briefly, a size exclusion chromatography-triple detector array (SEC-TDA) instrument consisting of two gel-permeation columns set in series (TSK-GEL GMPWXL, 7.8 × 30.0 cm, Tosoh Bioscience, Tokyo, Japan), a refractive index detector (RI), a laser detector made of a right-angle and a low-angle light scattering (RALS and LALS, respectively), and a four-bridge viscometer (VIS) was employed. A refractive index increment (d*n*/d*c*) value of 0.138 mL/g, as reported for water solutions of native, sulfated, or selenylated polymannuronate derivatives,^25^ was used for calculations. It is worth noting that the molecular weight data from SEC-TDA varied less than 10% when using 0.13–0.15 mL/g d*n*/d*c*, which is the range reported for unmodified and sulfated alginates (M-rich, G-rich, and unenriched species).^25−28^ An appropriate amount of each AS sample was dissolved in pure water and stirred at 25 °C overnight. The solution was then centrifuged, filtered on 0.22 μm filters, and analyzed for hydrodynamic characterization in triplicate. For each AS sample, molecular weight (weight average molar mass—*M*_w_, numeric average molar mass—*M*_n_, and polydispersity index—*M*_w_/*M*_n_), molecular size (hydrodynamic radius—*R*_h_), and intrinsic viscosity ([η]) distributions were derived. Due to their low molecular weight, tested samples behave as isotropic scatterers; therefore, only *R*_h_ could be derived as a measure of sample molecular size.

## Results and Discussion

**Scheme 1:**
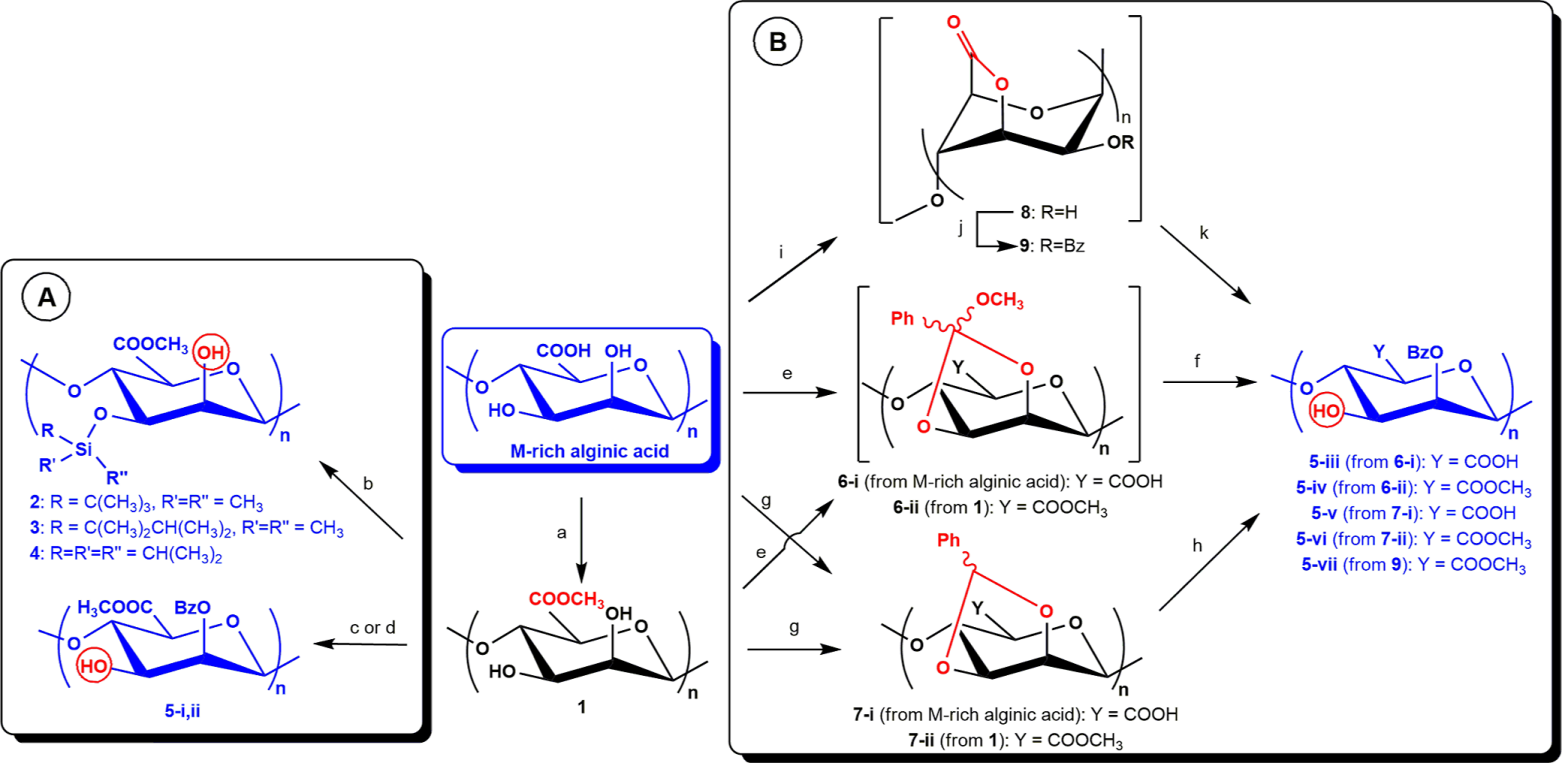
Different Strategies for the Regioselective Protection of M-rich Alginic Acid: (A) Direct Regioselective Protection of 2,3-Diols or (B) Installation and Regioselective Cleavage of Cyclic Protecting Groups ^a^H_2_SO_4_, CH_3_OH, 60 °C; ^b^TBDMSCl (or TDMSCl, or TIPSCl), imidazole, DMF, 50 °C; ^c^Bz_2_O, DIPEA, DMF, rt; ^d^BzCN, DMAP, 9:1 v/v DMF-pyridine, rt; ^e^PhC(OCH_3_)_3_, CSA, DMF, rt; ^f^9:1 v/v AcOH-H_2_O, rt; ^g^PhCH(OCH_3_)_2_, CSA, DMF, 80 °C; ^h^NaBrO_3_, Na_2_S_2_O_4_, H_2_O-ethyl acetate, rt; ^i^Bz_2_O, DMF, 85 °C; ^j^DMAP, pyridine, rt; ^k^NaOAc, CH_3_OH, rt.

Regioselective protection of the equatorial hydroxyl at 3-*O*-positions was first attempted by screening medium-size trialkylsilyl ether protecting groups (*t*-butyl-dimethylsilyl, TBDMS; thexyldimethylsilyl, TDMS; and triisopropylsilyl, TIPS), as they have been already employed for the selective monoprotection of the equatorial, less hindered hydroxyl of 2,3-diols in manno-configured monosaccharide derivatives,^29^ and/or for the differentiation of secondary hydroxyls in polysaccharides.^30^ The typical use of a slightly basic compound, such as imidazole, in combination with trialkylsilyl chlorides for hydroxyl silylation, suggested performing the protection reaction not on M-rich alginic acid itself but on its methyl ester derivative **1** (Scheme 1A), which could be obtained from the former by treatment with H_2_SO_4_ in dry methanol at 60 °C.^31^ Typical signals for TBDMS and TIPS ether groups could be detected at δ 0.8–0.0 and 1.0 ppm, respectively, in the ^1^H NMR spectrum of derivatives **2** and **4** (see Figures S2 and S4 in Supporting Information), whereas no significant signals related to TDMS ethers could be found in the ^1^H NMR spectrum of **3**, which was therefore not considered further.

Regioselective protection of the axial hydroxyl at 2-O-positions was tackled with two different approaches. The former relied upon two different organocatalyzed, site-selective methods reported for the insertion of benzoyl (Bz) esters preferentially on the axial position of cis-1,2-diols in pyranosides. They employ either Bz_2_O and DIPEA^32^ or BzCN and DMAP.^33^ These procedures were adapted to our polysaccharide case, giving derivatives **5-i,ii** from **1**, both showing ^1^H NMR spectra with characteristic signals for benzoylated species in the aromatic region and at chemical shifts between δ 4.8 and 5.5 ppm (see Figures S5 and S6 in Supporting Information).

An alternative strategy toward 2-*O*-benzoylated alginate was based on M-2,3-diol protection with cyclic protecting groups (CPGs) followed by the regioselective opening of the latter (Scheme 1B). Notably, CPGs are rarely employed on polysaccharides,^34−37^ although their use is very common in synthetic mono- and oligosaccharide chemistry.^38^ In particular, an orthobenzoate ring was first selected, and its installation was attempted on both native M-rich alginic acid and **1**, using PhC(OCH_3_)_3_ in DMF in the presence of CSA as the catalyst. Due to the high lability of cyclic orthoester moieties, the isolation of derivatives **6-i,ii** was not attempted. Instead, they were reacted in the same vessel^39^ with aqueous acetic acid, which is known to cleave the CPG and restore the alcohol moiety exclusively at equatorial sites, with the axial positions protected as Bz esters.^40^^1^H NMR spectra of the obtained derivatives **5-iii,iv** (see Figures S7 and S8 in Supporting Information) confirmed the presence of Bz esters, as demonstrated by the signals at δ 8.1–7.5 ppm related to aromatic hydrogen atoms together with signals at δ 5.2–5.7 ppm assignable to ring protons downfield shifted by an electron withdrawing group as Bz ester.

As an alternative to orthoester CPGs, benzylidene five-membered rings installed on 1,2-cis-diols of pyranosides can be opened under oxidative conditions to restore a free hydroxyl on the equatorial position of the diol and leave the axial oxygen atom protected as a Bz ester.^41^ Therefore, benzylidene rings were installed on M-rich alginic acid and its methyl ester derivative **1** by their treatment with PhCH(OCH_3_)_2_ in DMF in the presence of CSA as the acid catalyst. Unlike orthoester-type CPGs, derivatives carrying benzylidene rings are usually stable enough to be isolated. Indeed, derivatives **7-i,ii** were purified from the reaction mixture by precipitation, and the presence in their ^1^H NMR spectra of signals at δ 7.6–7.2 ppm associated with aromatic hydrogen atoms, together with signals at δ 6.1–5.7 ppm assignable to acetal hydrogen atoms downfield shifted by the adjacent phenyl ring (see Figures S11 and S12 in Supporting Information), demonstrated the formation of benzylidene rings. Their subsequent, oxidative opening with NaBrO_3_ and Na_2_S_2_O_4_ in water-ethyl acetate biphasic mixture afforded derivatives **5-v,vi**, displaying ^1^H NMR spectra (see Figures S9 and S10 in Supporting Information) with signal patterns similar to those detected for **5-iii,iv** samples after the orthoester installation/cleavage sequence.

A different CPG approach, based on the protection of 3,6-hydroxyacid moieties instead of 2,3-diols was also attempted through a three-step, one-pot sequence very recently developed on unsulfated chondroitin.^42^ This relies on the installation of a 3,6-lactone ring to link together carboxylic acid and OH-3 moieties.^43^ Interestingly, the intra-residue lactonization of alginic acid has been already reported some years ago through thermal dehydration under vacuum^44^ or by reaction with carboxylic acid activating agents, such as 2-chloro-1-methylpyridinium iodide (CMPI) or propylphosphonic anhydride (T3P),^45^ obtaining a degree of derivatization estimated to be far from quantitative. The lactonization reaction was instead performed here with Bz_2_O in DMF at 85 °C,^46^ followed by one-pot addition of DMAP and pyridine to protect the unreacted hydroxyls as benzoate esters and then of sodium acetate in methanol to selectively cleave the labile lactone and restore an alcohol moiety exclusively at position *C*-3. Unfortunately, the obtained derivative **5-vii** cannot be subjected to NMR analysis as it was found to be insoluble in any deuterated solvent.

**Scheme 2:**
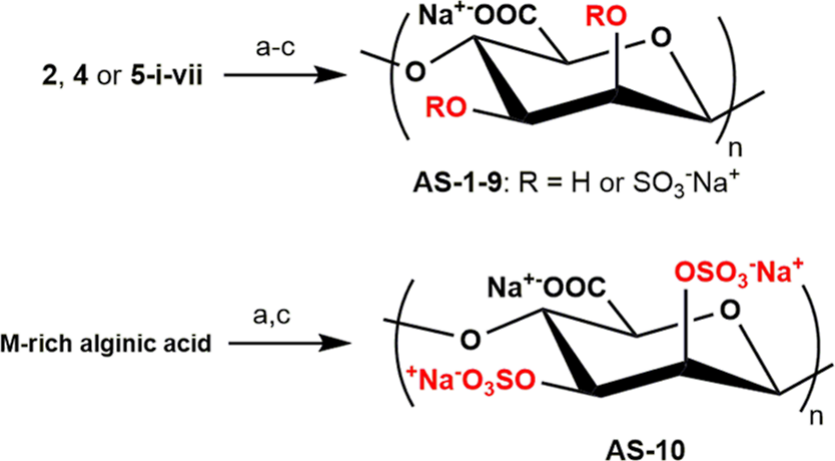
Semi-synthesis of Sulfated Alginates **AS-1-10** from M-rich Alginic Acid and Partially Protected Derivatives Thereof ^a^SO_3_·py, DMF, 50 °C; ^b^aq. H_2_SO_4_ (pH ∼ 2), 50 °C; ^c^aq. NaOH (pH ≥ 12), rt.

Derivatives **2**, **4**, and **5-i-vii** were subjected to sulfation under standard conditions^20^ with SO_3_·py in DMF at 50 °C, followed by a global deprotection under aqueous, slightly acid conditions to remove acid-labile TBDMS and TIPS ethers and any residual presence of benzylidene and orthoester CPGs and then by an alkaline treatment to hydrolyze methyl and benzoate esters (Scheme 2). In order to facilitate the structural study of the semi-synthetic sulfated alginates **AS-1-9**, a persulfated derivative was also obtained as a comparative standard by subjecting M-rich alginic acid to a direct sulfation under the same conditions employed on **2**, **4**, and **5-i-vii**.

Structural characterization of sulfated polysaccharides **AS-1-10** was conducted with a combination of several techniques. First, their hydrodynamic parameters were evaluated by high-performance size-exclusion chromatography combined with a triple detector array (HP-SEC-TDA). Molecular weight data were compared to the theoretical values expected for each sample, as calculated based on the experimental degree of sulfation (see below). All the semi-synthesized polysaccharides but two (**AS-3,8**) showed comparable molecular weights (Table 1), thus indicating that no significant depolymerization occurred during chemical modification. However, it is worth underlining that a low molecular weight (9 kDa) M-rich alginate sample was employed as the starting material due to the adverse effects (e.g., platelet aggregation and bleeding) typically associated with high molecular weight sulfated species and that the use of higher molecular weight alginate polysaccharides could affect depolymerization to a greater extent.

**Table 1:** Global Yield, Theoretical Molecular Weight, and Hydrodynamic Parameters for Semi-Synthetic Polysaccharides **AS-1-10**

	semi-synthetic intermediate	global mass yield^a^ (%)	global molar yield^b^ (%)	theoretical *M*_w_ (kDa)^c^	*M*_w_ (kDa)	*M*_w_/*M*_n_	[η] (dL/g)	*R*_h_ (nm)
**M-richalginic acid**					9 ± 2	1.3 ± 0.3	0.14 ± 0.01	2.4 ± 0.1
**AS-1**	**2**	11	7	13 ± 1	15 ± 6	1.5 ± 0.5	0.15 ± 0.03	3.0 ± 0.6
**AS-2**	**4**	29	20	14 ± 1	11 ± 5	2.0 ± 0.5	0.12 ± 0.03	2.4 ± 0.4
**AS-3**	**5-i**	71	42	13 ± 1	7 ± 2^d^	1.5 ± 0.2	0.10 ± 0.04	1.8 ± 0.4
**AS-4**	**5-ii**	13	11	n.d.^e^	n.d.^e^	n.d.^e^	n.d.^e^	n.d.^e^
**AS-5**	**5-iii**	121	64	15 ± 1	12 ± 5	1.5 ± 0.3	0.11 ± 0.01	2.5 ± 0.4
**AS-6**	**5-iv**	51	27	15 ± 1	10 ± 4	1.7 ± 0.2	0.12 ± 0.02	2.3 ± 0.3
**AS-7**	**5-v**	53	26	16 ± 1	11 ± 2	1.5 ± 0.1	0.14 ± 0.01	2.8 ± 0.1
**AS-8**	**5-vi**	15	9	13 ± 1	3 ± 1^d^	1.5 ± 0.3	0.06 ± 0.02	1.2 ± 0.2
**AS-9**	**5-vii**	134	62	17 ± 1	14 ± 4	1.5 ± 0.3	0.11 ± 0.01	2.7 ± 01
**AS-10**		181	83	17 ± 1	11 ± 3	1.4 ± 0.1	0.11 ± 0.01	2.5 ± 0.2

a Evaluated as the percentage ratio between the mass of the semi-synthetic AS derivative and the mass of the starting M-rich alginic acid (values higher than 100% are consistent due to an increase in molecular weight caused by sulfate group introduction).

b Evaluated as a percentage ratio between the repeating unit moles obtained for the semi-synthetic AS derivative as sodium salt and the repeating unit moles of the starting M-rich alginic acid. The calculation took into consideration the average value of DS estimated by NMR and by elemental analysis (see Table 2) by applying eq 2 (*y*_molar_ = global molar yield, *y*_mass_ = global mass yield, *m*_W(AA)_ = M-rich alginic acid repeating unit molecular weight = 176, *m*_W(ANa)_ = M-rich sodium alginate repeating unit molecular weight = 198, and Δ*m*_W_ = molecular weight gain due to a single sodium sulfate group installation = 102).

*y*_molar_ = {*y*_mass_/[*m*_W(ANa)_ + (Δ*m*_W_ × ⟨DS⟩)]}/(1/*m*_W(AA)_) (2)

c Calculated taking into consideration the average value of DS estimated by NMR and by elemental analysis (see Table 2), by applying eq 3 (*M*_w_^M-rich^ = experimental *M*_w_ for starting M-rich alginic acid = 9 ± 2).

*M*_w_^theoretical^ = *M*_w_^M-rich^{[*m*_W(ANa)_ + Δ*m*_W_ × ⟨DS⟩]/*m*_W(ANa)_} (3)

d *p* < 0.05 compared to the theoretical molecular weight.

e Not determined.

Concerning the presence of sulfur atoms in **AS-1-10**, it was confirmed by elemental analysis data, which also allowed the evaluation of DS^47^ (Table 2). In order to gain insights on sulfate group distribution on **AS-1-10** backbones, 2D-NMR spectra were recorded for these derivatives as well as for starting M-rich alginic acid. ^1^H- and ^13^C NMR chemical shifts of both unsulfated and persulfated (**AS-10**) M-rich alginic acid spectra (see Figures S13 and S29 in Supporting Information) were found to be in agreement with literature values.^22,48^

**Table 2:** Structural Features of Semi-synthetic Polysaccharides **AS-1-10**

			degree of sulfation^b^			
	DS (El. Anal.)^a^	DS (NMR)^b^	M^c^	M2S^c^	M3S^c^	M2S3S^c^
**M-richalginic acid**	0.03 ± 0.01		100%			
**AS-1**	0.84 ± 0.01	1.0	20%	60%	n.d.^d^	20%
**AS-2**	1.01 ± 0.01	1.16	14%	52%	2%	32%
**AS-3**	0.81 ± 0.11	1.19	3%	n.d.^d^	75%	22%
**AS-4**	0.17 ± 0.03	n.d.^d^	n.d.^d^	n.d.^d^	n.d.^d^	n.d.^d^
**AS-5**	1.32 ± 0.12	1.37	n.d.^d^	43%	20%	37%
**AS-6**	1.20 ± 0.02	1.41	n.d.^d^	31%	28%	41%
**AS-7**	1.55 ± 0.01	1.63	n.d.^d^	25%	12%	63%
**AS-8**	0.81 ± 0.12	0.96	4%	51%	45%	n.d.^d^
**AS-9**	1.75 ± 0.03	1.88	n.d.^d^	12%	n.d.^d^	88%
**AS-10**	1.81 ± 0.01	1.87	n.d.^d^	13%	n.d.^d^	87%

a Evaluated by elemental analysis data.

b Estimated by ^1^H,^13^C-HSQC NMR signal volume integration.

c M denotes unsulfated ManA units. Data expressed as relative percentages with respect to the total amount of M units.

d Not detected.

**Figure 2:**
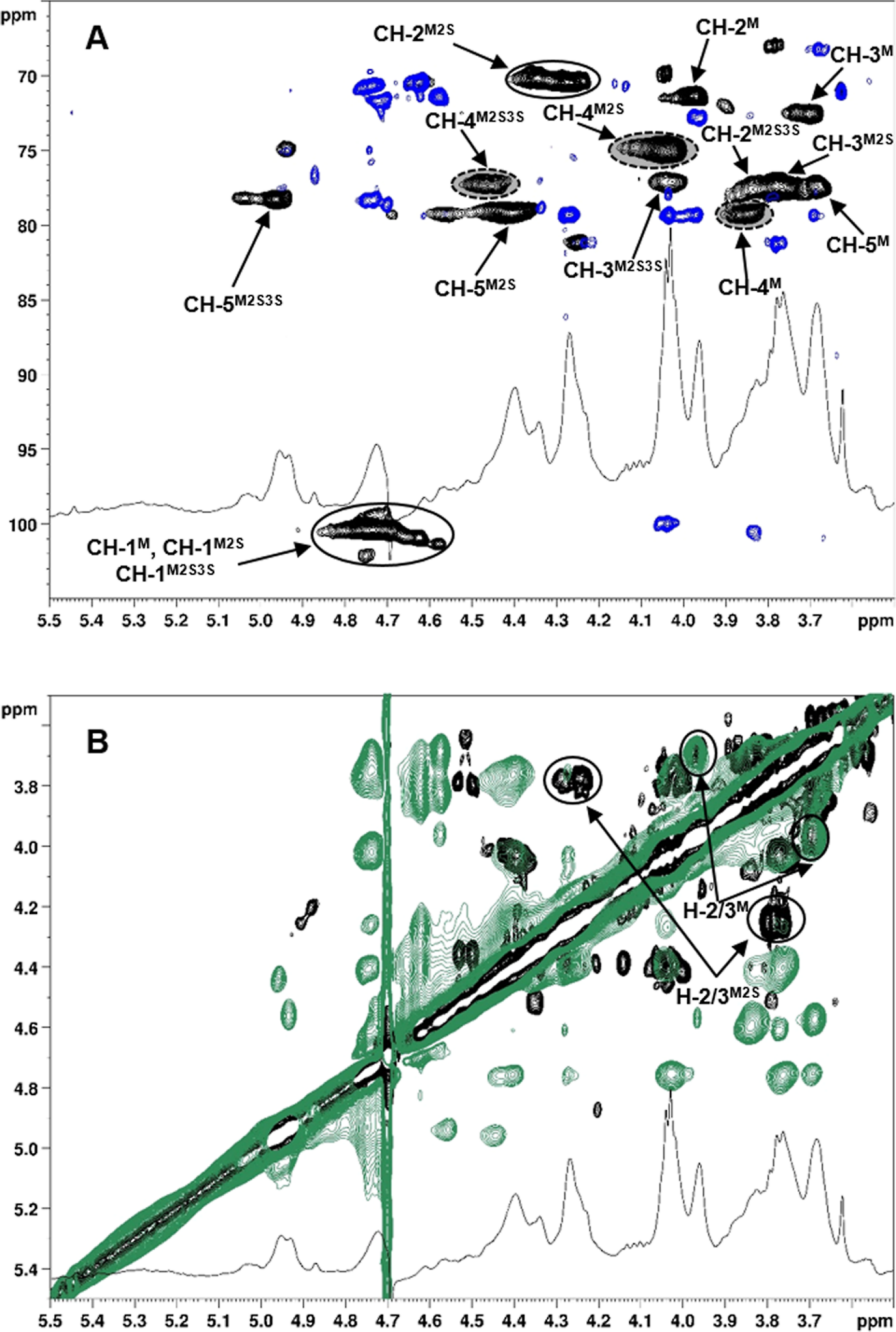
Superimposition of zoomed (A) ^1^H NMR, ^1^H,^13^C-HSQC (black), and ^1^H,^13^C-HMBC (blue) 2D-NMR (densities enclosed in dotted lines were integrated for DS estimation) and (B) ^1^H NMR, COSY (black), and NOESY (green) 2D-NMR spectra (600 MHz, 298 K, D_2_O) of **AS-1** (only some correlations are indicated).

The NMR spectra of the derivative **AS-1** were then analyzed. The superimposition of ^1^H,^13^C-HSQC and ^1^H,^13^C-HMBC (Figure 2A) spectra allowed the assignment of two groups of CH signals in the ^1^H,^13^C-HSQC spectrum to M-2 positions, at δ_H/C_ 3.98/71.4 and 4.35–4.23/70.0–70.6 ppm, respectively, from their HMBC correlation with anomeric CH signals. By comparison with the ^1^H,^13^C-HSQC spectrum of starting M-rich alginic acid (see Figure S16 in Supporting Information), the former CH-2 signal could be assigned to unsulfated M units. This was confirmed by its HMBC and COSY (Figure 2B) correlation with the signal at 3.71/72.6 ppm, associated with CH-3 of unsulfated M units. The downfield shift of the CH-2 signal at δ_H/C_ 4.35–4.23/70.0–70.6 ppm was due to the presence of an electron-withdrawing sulfate group at the O-2 site. Its COSY correlation with a signal at δ 3.78 ppm allowed the recognition of the M2S unit, as no downfield shift caused by sulfation was detected for the CH-3 signal. Moreover, the most ^1^H-downfield-shifted signal in the ^1^H,^13^C-HSQC spectrum at δ_H/C_ 4.96/77.1 ppm was indicative of the presence of additional sulfate groups at position 3 of some M units. The discrimination between M3S and 2,3-disulfated (M2S3S) units was possible on the basis of the presence or absence (as in this case), respectively, of the typical signal for M3S CH-3 atoms at δ_H/C_ 4.73/79.3 ppm (see below). Therefore, the contemporary presence of unsulfated M, M2S, and M2S3S units along the polysaccharide chain could be inferred for **AS-1**.

The relative integration in the ^1^H,^13^C-DEPT-HSQC spectrum of the well-resolved CH-4 signal volumes at δ_H/C_ 3.84/78.9, 4.03/74.9, and 4.47/77.1 ppm, which could be assigned to M, M2S, and M2S3S units, respectively, allowed estimating a 20%:60%:20%: ratio for M, M2S, and M2S3S residues in **AS-1**, assuming that the three CH-4 signals displayed similar ^1^*J*_C,H_ coupling constants and that a difference of around 5–8 Hz from the experimental set value did not cause in any case a substantial variation of the integrated peak volumes.^49,50^ The relative M/M2S/M2S3S ratio estimated by 2D-NMR analysis returned a DS equal to 1.0 for **AS-1** (Table 2), roughly supporting that measured by elemental analysis (DS = 0.84 ± 0.02).

A similar analysis of 2D-NMR spectra was conducted on derivative **AS-2** (see Figures S17 and S18 in Supporting Information), confirming rough agreement between DS values calculated from elemental analysis data (DS = 1.01 ± 0.01) and estimated by 2D-NMR integration (DS = 1.16), but also revealing the presence of a lower relative amount of the target M2S residues (52%) with respect to **AS-1** and a higher amount of M2S3S units (32%). Therefore, a slightly higher regioselectivity could be inferred for the semi-synthetic strategy for a 2-O-sulfated polysaccharide derivative employing TBDMS vs TIPS ether protecting groups.

The ^1^H,^13^C-HSQC NMR spectra of derivatives **AS-3-9** were expected to show signals at different chemical shifts with respect to **AS-1,2**, especially for CH-2 and CH-3 atoms, due to the complementary, targeted regioselectivity of sulfate group installation. **AS-5,6** showed indeed two density volumes at δ_H/C_ 3.88/71.7 and 4.73/79.3 ppm, which were not detected among the significant signals in **AS-1,2** spectra. They could be assigned with the help of ^1^H,^13^C-HMBC and COSY NMR spectra (see Figures S21, S23, S25, and S26 in Supporting Information) to CH atoms at O-2 and O-3 positions, respectively, of M3S units. The ^1^H,^13^C-HSQC NMR spectra of **AS-5,6** revealed the presence of M2S and M2S3S residues too. The M2S/M3S/M2S3S relative amounts were estimated to be equal to 43%:20%:37% (for **AS-5**) and 31%:28%:41% (for **AS-6**), by relative integration of CH-3^M3S^ vs CH-3^M2S3S^ and CH-4^M2S^ vs CH-4^M2S3S^ signals. Such sulfation pattern data showed that the semi-synthetic strategies based on the orthoester protecting group were not useful to achieve the targeted regioselective sulfation at the O-3 site.

The other tested CPGs gave unsatisfying results as well. Indeed, the semi-synthetic strategy based on the intramolecular 3,6-lactone CPG afforded the derivative **AS-9**, for which the DS evaluation by both elemental analysis and ^1^H,^13^C-HSQC 2D-NMR spectra integration (see Figure S28 in Supporting Information) revealed an almost quantitative per-O-sulfation. This suggested that the one-pot 3,6-lactonization/benzoylation/lactone opening sequence was almost entirely unable to protect the hydroxyls of the M-rich alginic acid chain. Neither was regioselective sulfation detected for **AS-7,8**. It was noteworthy that, while orthoester CPG-derived products **AS-5,6** gave similar DS and sulfation patterns, regardless of the employed starting material (M-rich alginic acid or its methyl ester derivative **1**), a marked difference was found for **AS-7,8**, derived by the use of a benzylidene CPG on the same precursors. In particular, the semi-synthetic strategy developed from M-rich alginic acid afforded the derivative **AS-7**, which has M-2,3S as a major unit (63%) along the polysaccharide chain, while in **AS-8** derivative—obtained starting from methyl ester **1**—the same disulfated residue was not detected at all. This marked difference could be presumably ascribed to a different behavior of benzylidene-protected intermediates **7-i** and **7-ii** in the CPG ring opening reaction with NaBrO_3_ and Na_2_S_2_O_4_ in a water-ethyl acetate biphasic mixture (Scheme 1). The reaction on **7-ii** furnished derivative **5-vi**, that was composed almost exclusively of mono-Bz ester protected M units, deriving from the expected bromine radical-mediated oxidative mechanism,^41^ although the targeted regioselective protection at the O-2 position was not achieved, as highlighted by the very similar amount estimated for M2S and M3S units in derivative **AS-8**. Conversely, competition by a hydrolytic mechanism was hypothesized for the benzylidene cleavage reaction on free acid derivative **7-i**, thus affording unprotected residues as major units in the polysaccharide chain of **5-v** and therefore explaining the predominance of disulfated M2S3S-units in derivative **AS-7**. This hydrolytic mechanism could be mediated by the carboxylic acid functionality of M units. Furthermore, different from derivative **7-ii**, partial solubility of **7-i** in the aqueous phase of the reaction mixture was observed (see Experimental Section). This could have allowed further activation of the benzylidene hydrolysis reaction through a preliminary electrophilic bromination of the aromatic ring by bromonium ions generated in situ in the water phase from a NaBrO_3_–Na_2_S_2_O_4_ mixture.^51^

**Figure 3:**
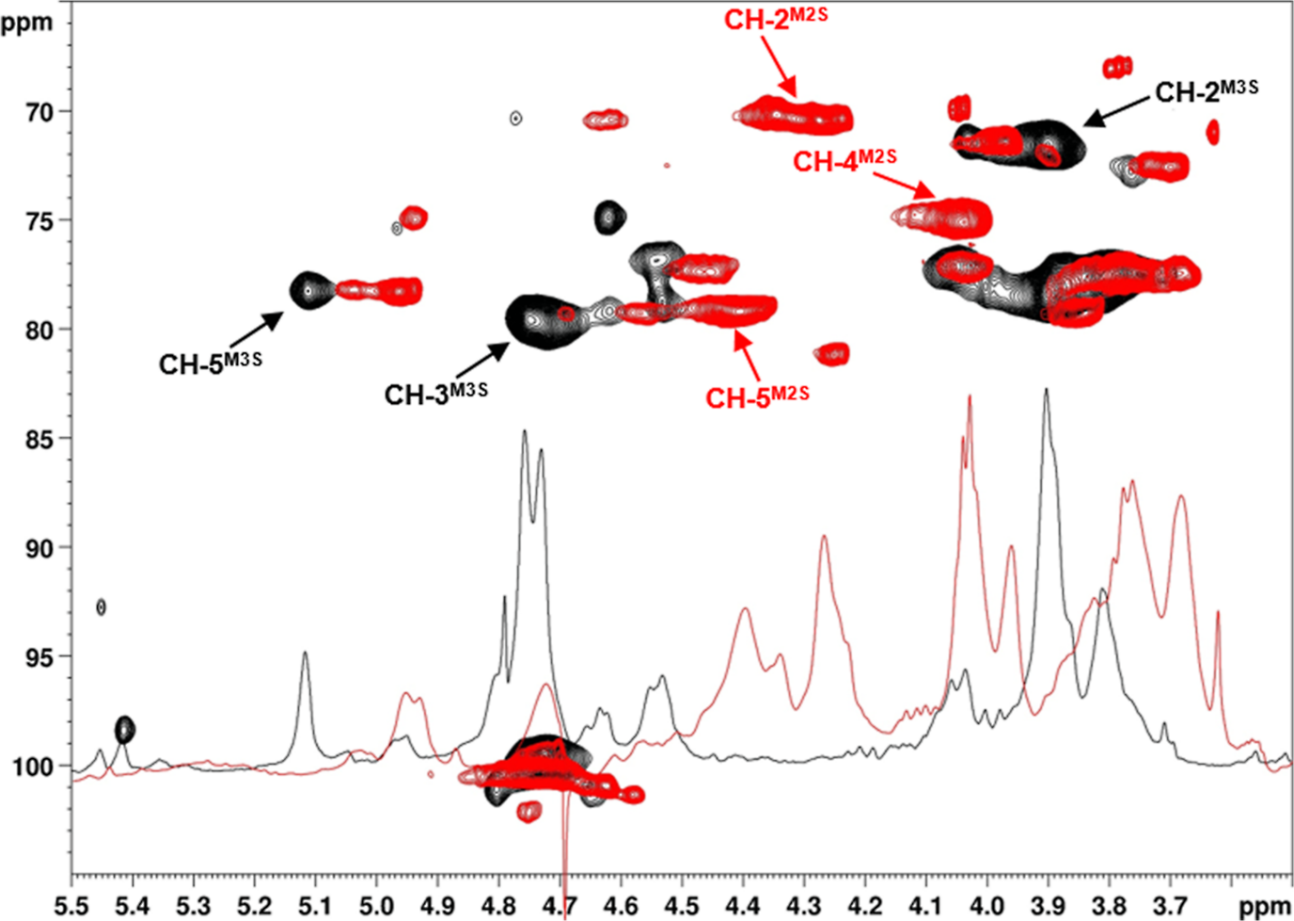
Superimposition of zoomed ^1^H NMR and ^1^H,^13^C-HSQC 2D-NMR spectra (600 MHz, 298 K, D_2_O) of **AS-1** (red) and **AS-3** (black) (the assignment only of the signals differing between the two derivatives is here indicated).

Much higher control of sulfation pattern was achieved for the derivative **AS-3**, obtained through regioselective protection of the methyl esterified M units at the O-2 position by DIPEA-promoted benzoylation with Bz_2_O. Its 2D-NMR spectra (see Figure S19 in Supporting Information) revealed a marked prevalence for M3S units (75%) along the polysaccharide backbone, together with a minor amount of M2S3S residues (22%). A superimposition of ^1^H- and ^1^H,^13^C-DEPT-HSQC NMR spectra of **AS-3** and **AS-1** (Figure 3) clearly confirmed the complementary positioning of sulfate groups (at O-3 or O-2 positions, respectively) on the majority of M units of each derivative. Conversely, derivative **AS-4**, obtained through a semi-synthetic strategy having as a key step the reaction of methyl ester derivative **1** with BzCN as benzoylating reagent, gave a ^1^H NMR spectrum very similar to the starting M-rich alginate one. This result was in agreement with the very low DS value (0.17) obtained by elemental analysis of **AS-4** and can be explained by an almost quantitative benzoylation at both the O-2 and O-3 positions in the semi-synthetic intermediate **5-ii** instead of the targeted regioselective protection at the axial site.

## Conclusions

Regioselective sulfation of equatorial *vs.* axial hydroxyls of M-rich alginic acid was attempted through several different multi-step strategies, all relying upon protection-sulfation-deprotection sequences. All the semi-synthesized sulfated polysaccharides were subjected to a detailed characterization of their sulfation pattern through a combination of elemental analysis, FT-IR, and 1D- and 2D-NMR spectroscopy.

A marked prevalence of sulfation at the O-2 vs O-3 sites was achieved through the employment of TBDMS ether to protect, regioselectively, the equatorial O-3 position, thereby allowing sulfation on the C-2 axial hydroxyl. A complementary sulfation pattern was accessed by protecting the O-2 position with Bz_2_O in the presence of DIPEA. Therefore, in this work, we have demonstrated that regioselectively sulfated derivatives of M-rich alginic acid can be accessed by properly tailored, multi-step semi-synthetic sequences. Application of the developed strategies to structurally different alginates (e.g., G-rich and non-G/M-enriched species and low- and high-molecular weight polysaccharides) is currently underway and could be reliable for other polysaccharides for which access to regioselectively sulfated species still remains elusive.^20^ The alginate sulfate species described here, together with the other ones currently under investigation, will allow detailed investigations of their structure–activity relationships and therefore shed new light on the molecular details of alginate sulfate properties and applications.

## Supporting Information Available

1D- and 2D-NMR spectra of starting M-rich alginic acid and semi-synthetic sulfated derivatives thereof (PDF)

## Abbreviations

Ac: acetyl
AS: alginate sulfate
Bz: benzoyl
CMPI: 2-chloro-1-methylpyridinium iodide
COSY: correlation spectroscopy
CPG: cyclic protecting group
CSA: d-camphor-10-sulfonic acid
DIPEA: *N*,*N*-diisopropylethylamine
DMAP: 4-(dimethylamino)pyridine
DMF: *N*,*N*-dimethylformamide
DMSO: dimethylsulfoxide
DS: degree of sulfation
EDTA: ethylenediaminetetraacetic acid
EtOH: ethanol
GulA, G: l-guluronic acid
HMBC: heteronuclear multibond correlation
HP: high-performance
HSQC: heteronuclear single quantum coherence
LALS: low-angle light scattering
ManA, M: d-mannuronic acid
M2S: 2-*O*-sulfated-d-mannuronic acid
M2S3S: 2,3-di-*O*-sulfated-d-mannuronic acid
M3S: 3-*O*-sulfated-d-mannuronic acid
*M*_n_: numeric average molar mass
*M*_w_: weight average molar mass
NMR: nuclear magnetic resonance
NOESY: nuclear Overhauser effect spectroscopy
PSS: propylene glycol alginate sodium sulfate
RALS: right-angle light scattering
*R*_h_: hydrodynamic radius
RI: refractive index
rt: room temperature
SAR: structure–activity relationships
SEC: size exclusion chromatography
SO_3_·py: pyridine–sulfur trioxide complex
T3P: propylphosphonic anhydride
TBDMS: *t*-butyl-dimethylsilyl
TDA: triple detector array
TDMS: thexyldimethylsilyl
TIPS: triisopropylsilyl
VIS: viscometer
[η]: intrinsic viscosity
