# Supplementary material for: Multi-step Strategies Toward Regioselectively Sulfated M-Rich Alginates
Source: Biomacromolecules. 2023 Apr 28;24(6):2522–31. doi: 10.1021/acs.biomac.3c00045 (PMC10265665; doi:10.1021/acs.biomac.3c00045)
Supplement: Supplementary file 1 — bm3c00045_si_001.pdf [file bm3c00045_si_001.pdf]

MULTI-STEP STRATEGIES TOWARDS REGIOSELECTIVELY SULFATED M-RICH ALGINATES

Supporting Information

FABIANA ESPOSITO,[1] ANTONIO LAEZZA,[2] SERENA TRABONI,[1]

ALFONSO IADONISI,[1] VALENTINA GARGIULO,[3] ANNALISA LA GATTA,[4]

CHIARA SCHIRALDI,[4] EMILIANO BEDINI[1]

[1]Department of Chemical Sciences, University of Naples Federico II,
Complesso Universitario Monte S.Angelo, via Cintia 4, I-80126 Napoli, Italy
[2]Department of Sciences, University of Basilicata, viale dell'Ateneo Lucano 10, I-85100 Potenza, Italy
[3] Institute of Sciences and Technologies for Sustainable Energy and Mobility,
National Research Council (STEMS-CNR), piazzale V. Tecchio 80, I-80125 Napoli, Italy
[4] Department of Experimental Medicine, Section of Biotechnology, University of Campania "Luigi Vanvitelli", via de Crecchio 7, I-80138 Napoli, Italy

Table of Contents

Figure S1: ^{1}H NMR spectrum of **1** S-3

Figure S2: ^{1}H-NMR spectrum of **2** S-3

Figure S3: ^{1}H-NMR spectrum of **3** S-4

Figure S4: ^{1}H-NMR spectrum of **4** S-4

Figures S5-S10: ^{1}H-NMR spectrum of **5i-vi** S-5-7

Figures S11-S12: ^{1}H-NMR spectrum of **7i-ii** S-8

Figure S13: ^{1}H, ^{1}H,^{13}C-HSQC and ^{1}H,^{13}C-HMBC NMR spectra of M-rich alginic acid S-9

Figure S14: ^{1}H, COSY and NOESY NMR spectra of M-rich alginic acid S-9

Figure S15: Zoomed ^{1}H NMR spectra of M-rich alginic acid S-10

Figure S16: ^{1}H and ^{1}H,^{13}C-HSQC NMR spectra superimposition of **AS-1** and M-rich alginic acid S-10

Figure S17: ^{1}H and ^{1}H,^{13}C-HSQC NMR spectra of **AS-2** S-11

Figure S18: ^{1}H and COSY NMR spectra of **AS-2** S-11

Figure S19: ^{1}H and ^{1}H,^{13}C-HSQC NMR spectra of **AS-3** S-12

Figure S20: ^{1}H NMR spectra superimposition of **AS-4** and M-rich alginic acid S-12

Figure S21: ^{1}H and ^{1}H,^{13}C-HSQC NMR spectra of **AS-5** S-13

Figure S22: ^{1}H and COSY NMR spectra of **AS-5** S-13

Figure S23: ^{1}H, ^{1}H,^{13}C-HSQC and ^{1}H,^{13}C-HMBC NMR spectra of **AS-6** S-14

Figure S24: ^{1}H and COSY NMR spectra of **AS-6** S-14

Figure S25: ^{1}H and ^{1}H,^{13}C-HSQC NMR spectra superimposition of **AS-6** and **AS-1** S-15

Figure S26: ^{1}H and ^{1}H,^{13}C-HSQC NMR spectra of **AS-7** S-15

Figure S27: ^{1}H and ^{1}H,^{13}C-HSQC NMR spectra of **AS-8** S-16

Figure S28: ^{1}H and ^{1}H,^{13}C-HSQC NMR spectra of **AS-9** S-16

Figure S29: ^{1}H and ^{1}H,^{13}C-HSQC NMR spectra of **AS-10** S-17

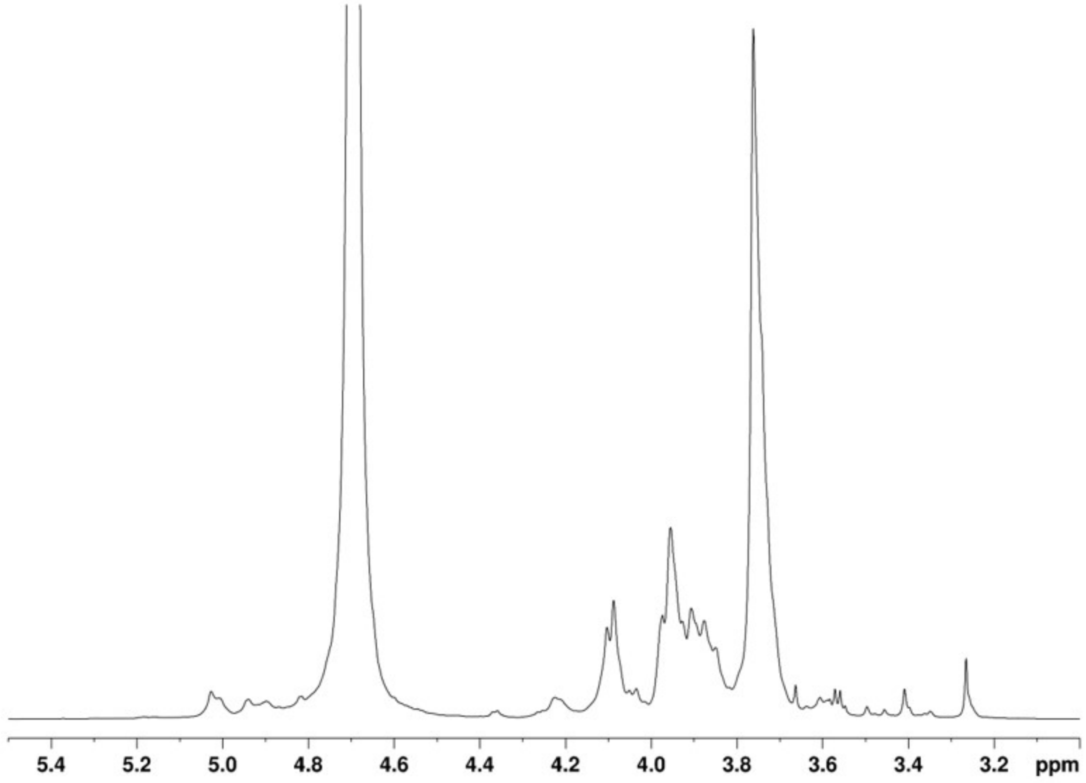

Figure S1: ^{1}H-NMR spectrum (600 MHz, 298K, D_2O) of **1**

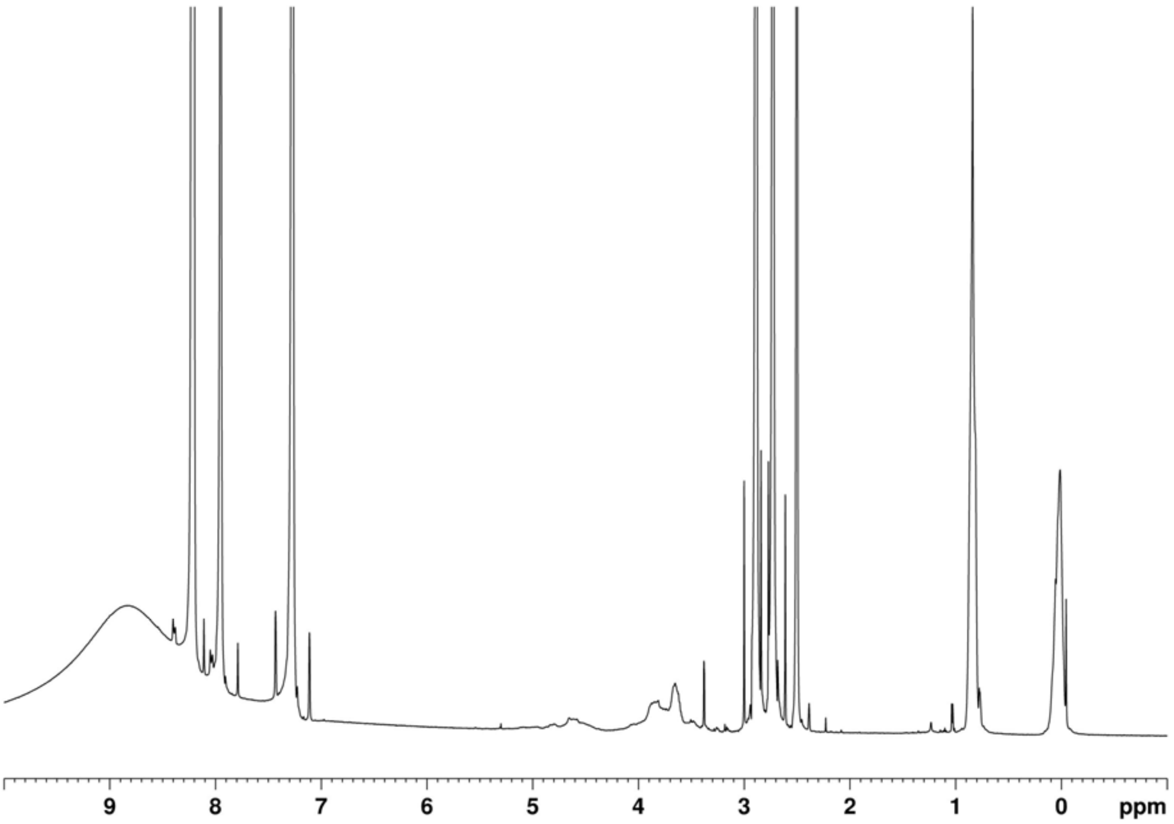

Figure S2: ^{1}H NMR spectrum (600 MHz, DMSO-d_6, 298 K) of **2**

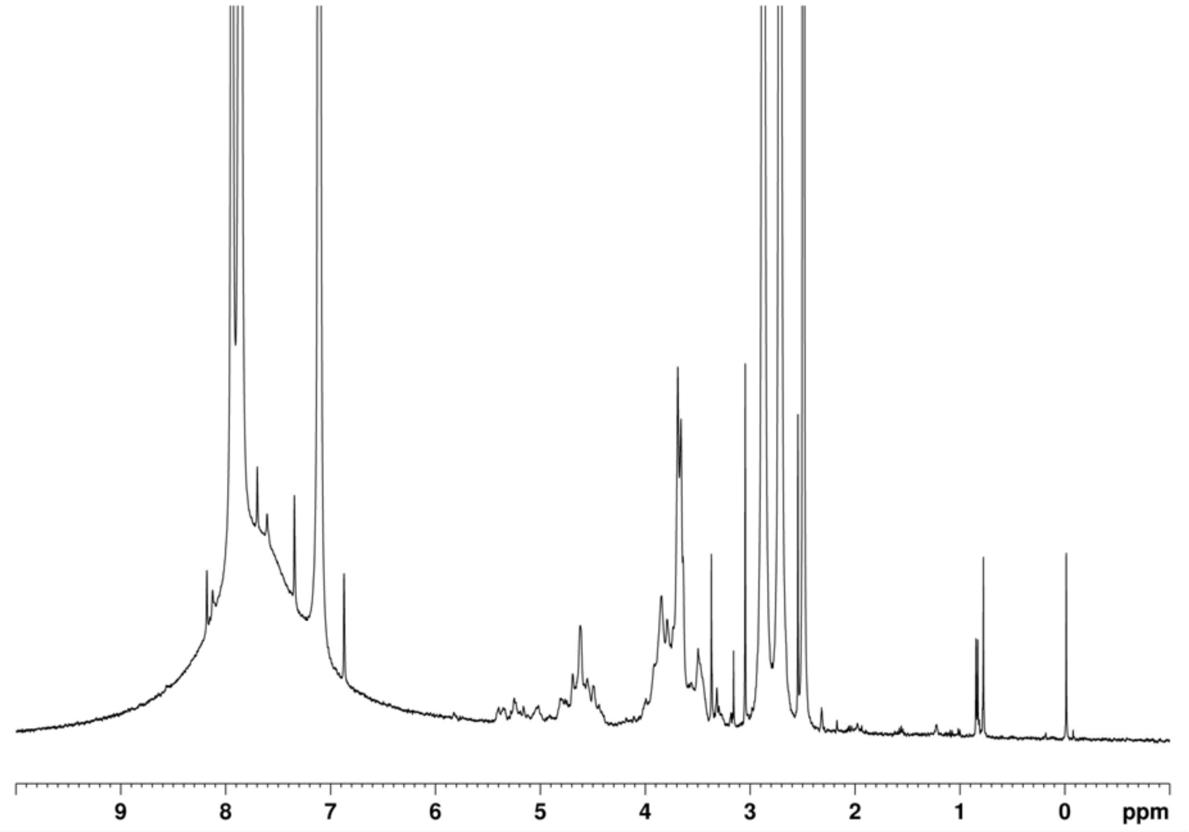

Figure S3: ^{1}H NMR spectrum (600 MHz, DMSO-d_6, 298 K) of **3**

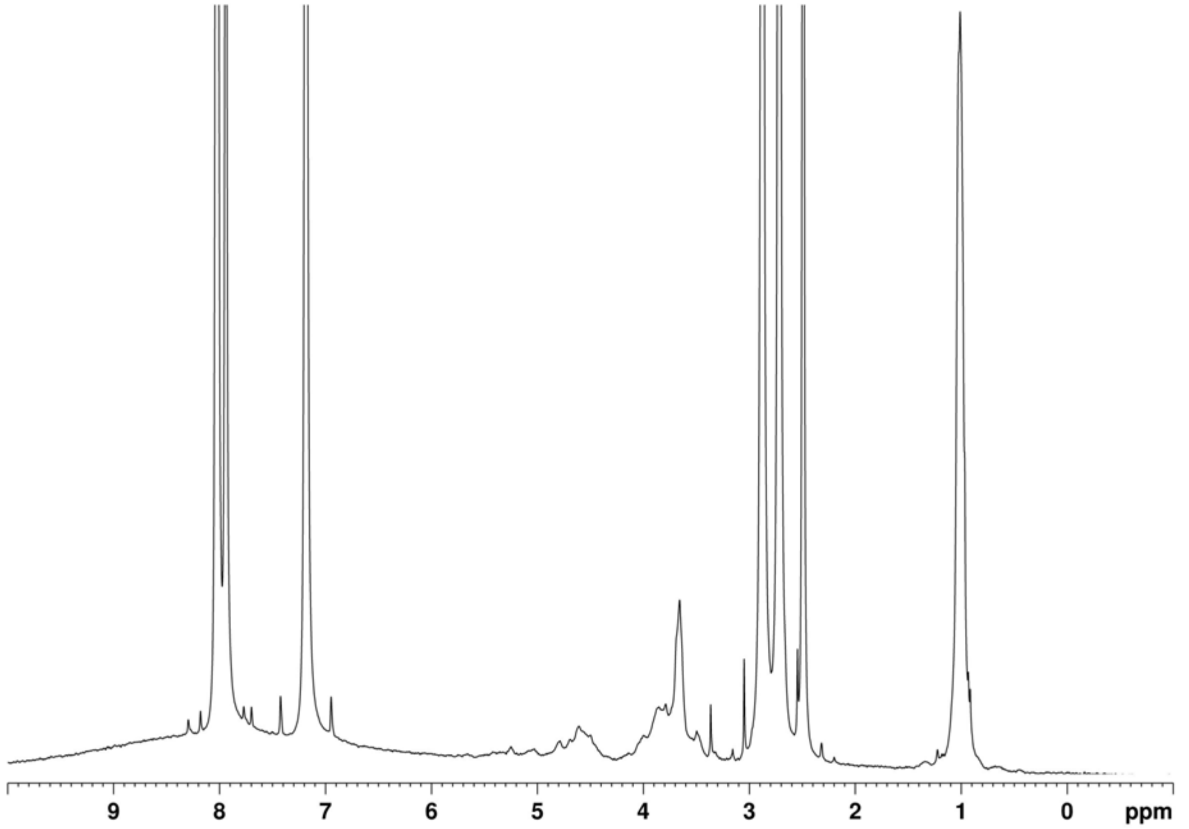

Figure S4: ^{1}H NMR spectrum (600 MHz, DMSO-d_6, 298 K) of **4**

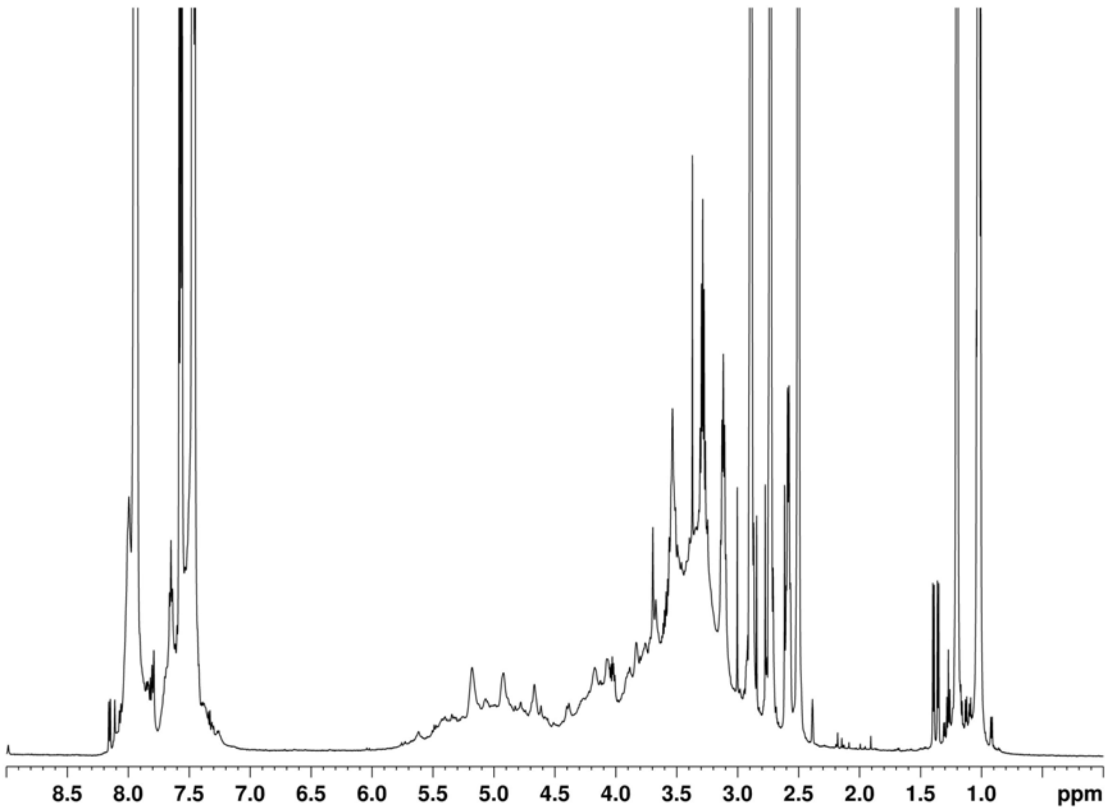

Figure S5: ^{1}H NMR spectrum (600 MHz, DMSO-d_6, 298 K) of **5-i**

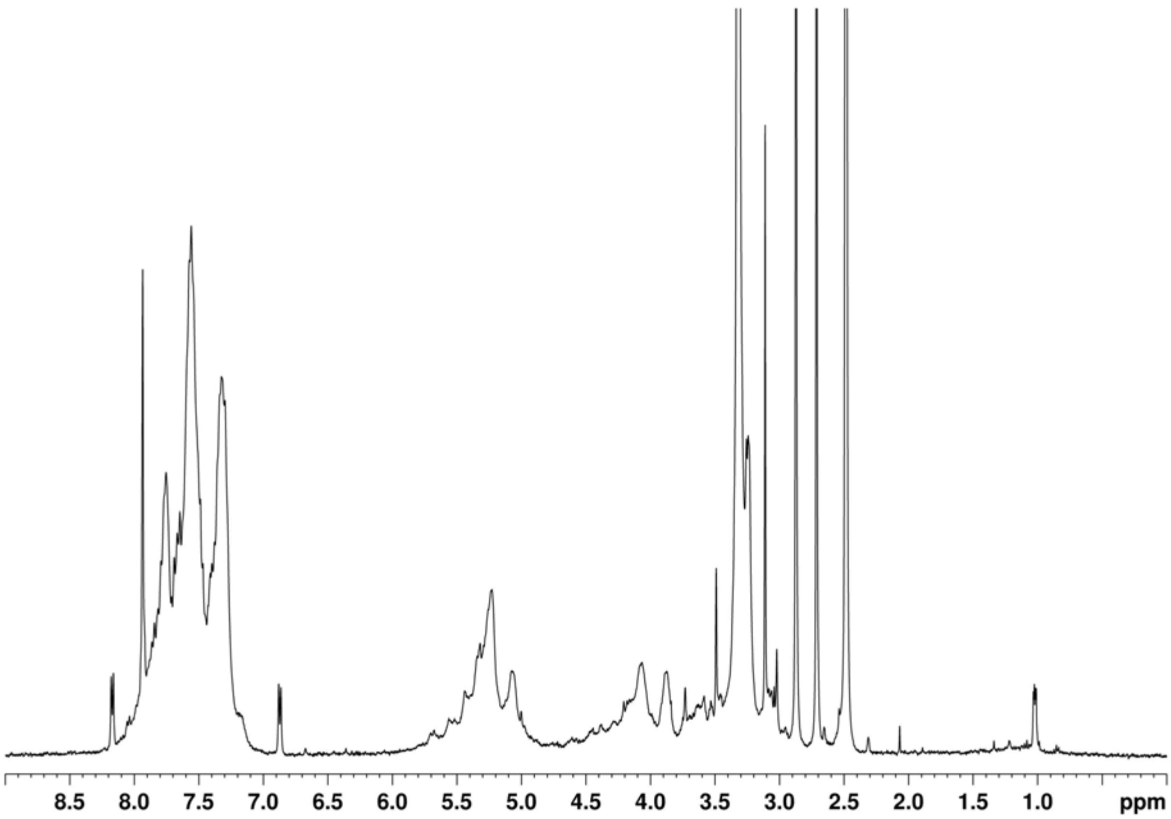

Figure S6: ^{1}H NMR spectrum (400 MHz, DMSO-d_6, 298 K) of **5-ii**

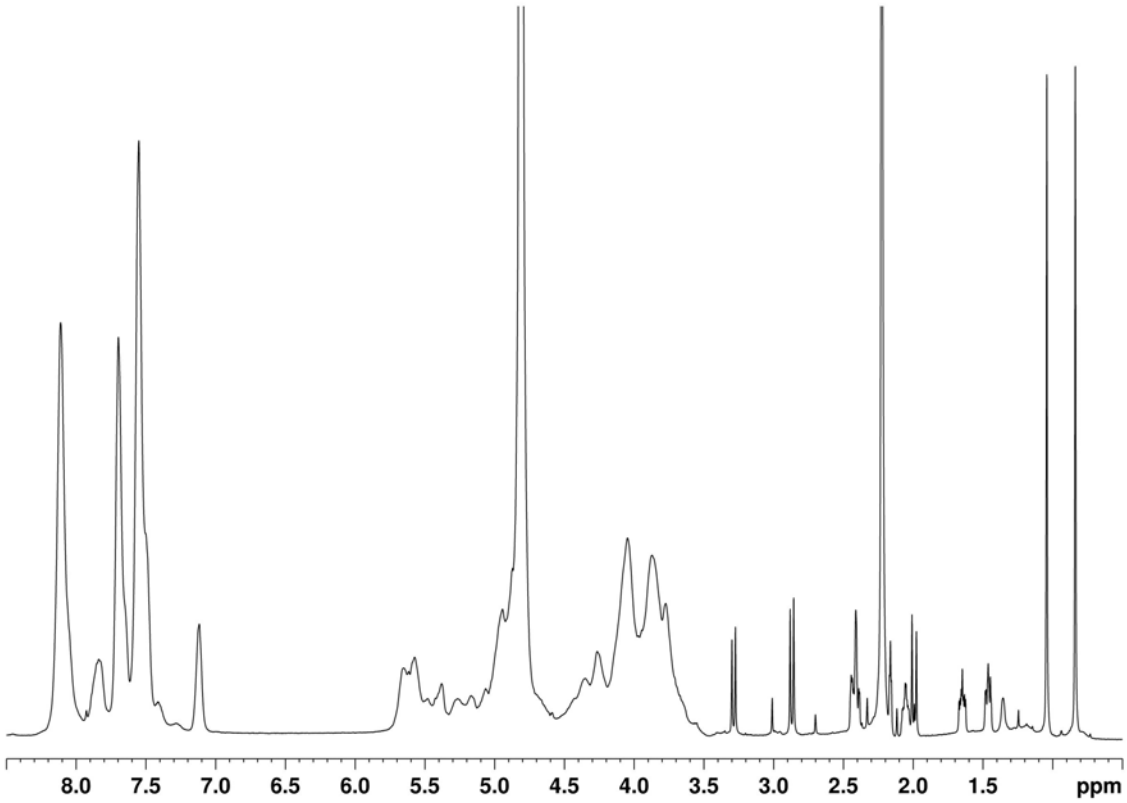

Figure S7: ^{1}H NMR spectrum (600 MHz, DMSO-d_6, 298 K) of **5-iii**

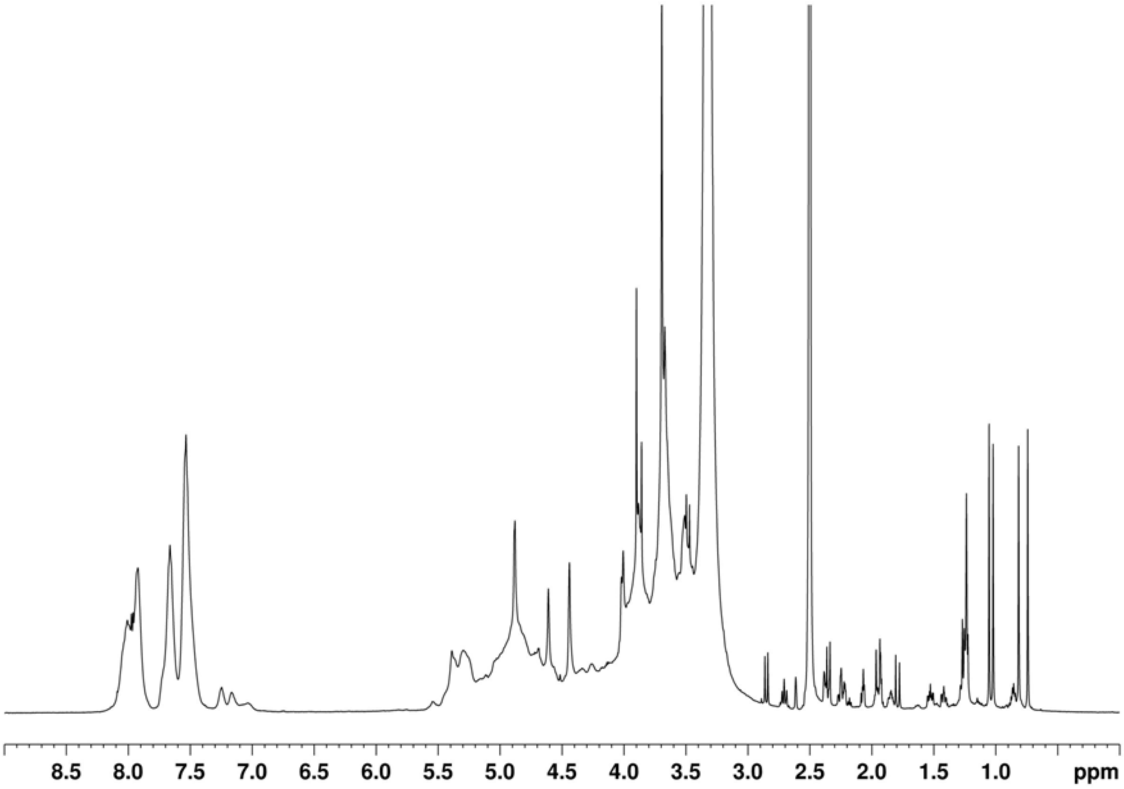

Figure S8: ^{1}H NMR spectrum (600 MHz, DMSO-d_6, 298 K) of **5-iv**

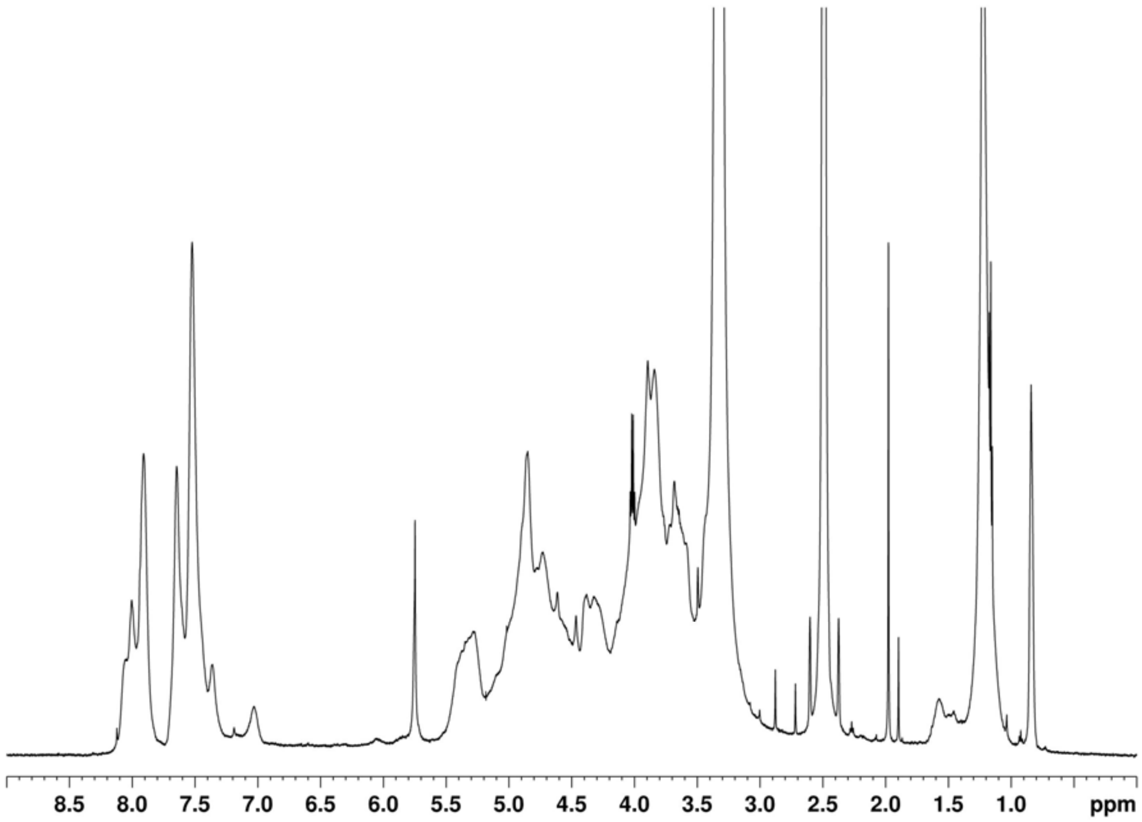

Figure S9: ^{1}H NMR spectrum (600 MHz, DMSO-d_6, 298 K) of **5-v**

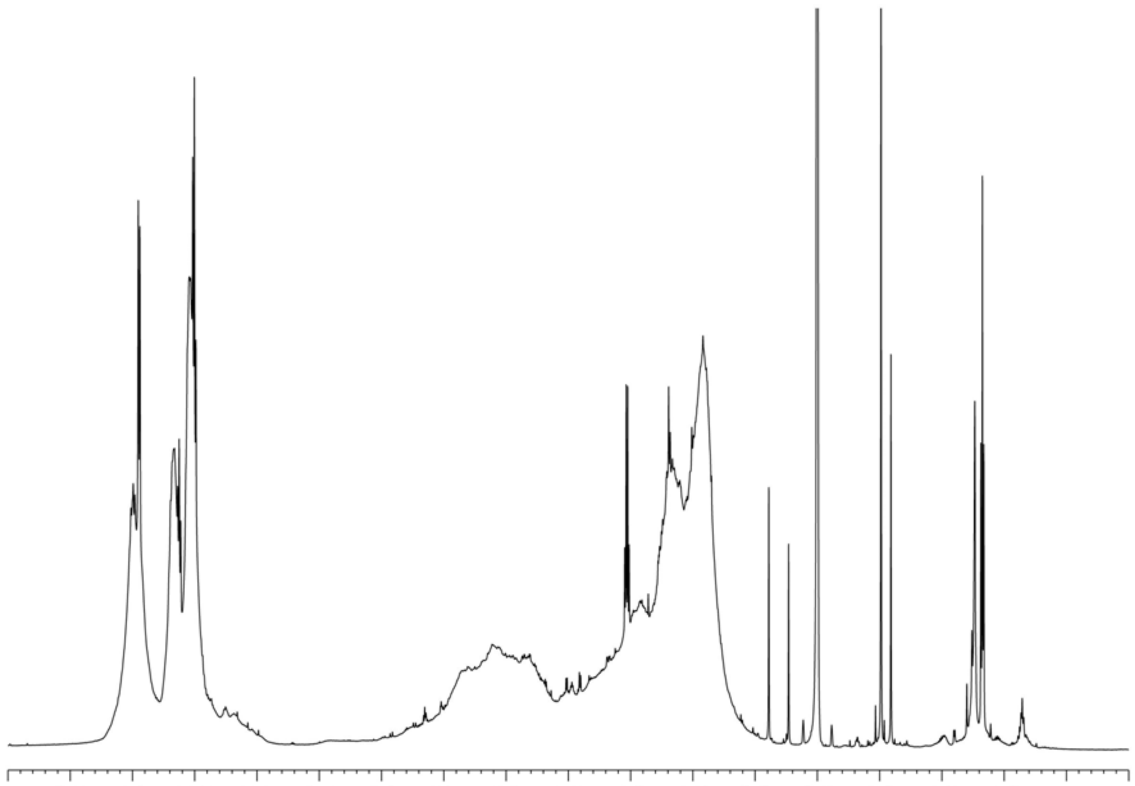

Figure S10: ^{1}H NMR spectrum (600 MHz, DMSO-d_6, 298 K) of **5-vi**

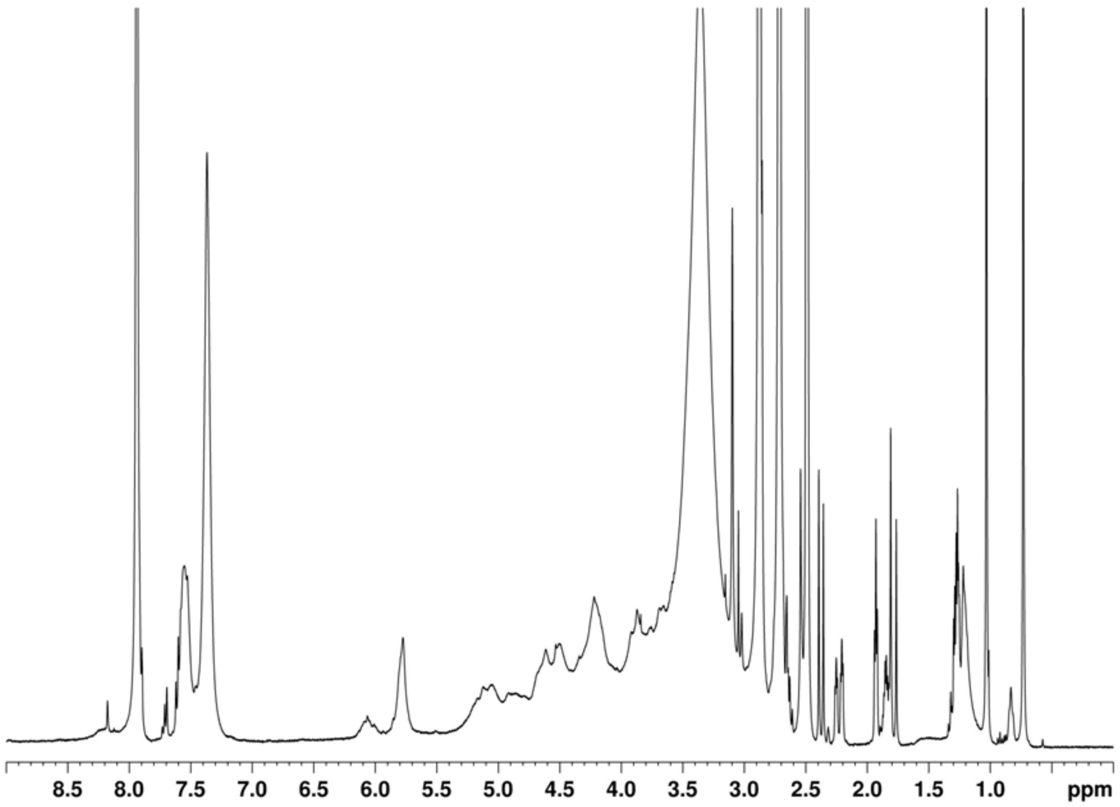

Figure S11: ^{1}H NMR spectrum (600 MHz, DMSO-d_6, 298 K) of **7-i**

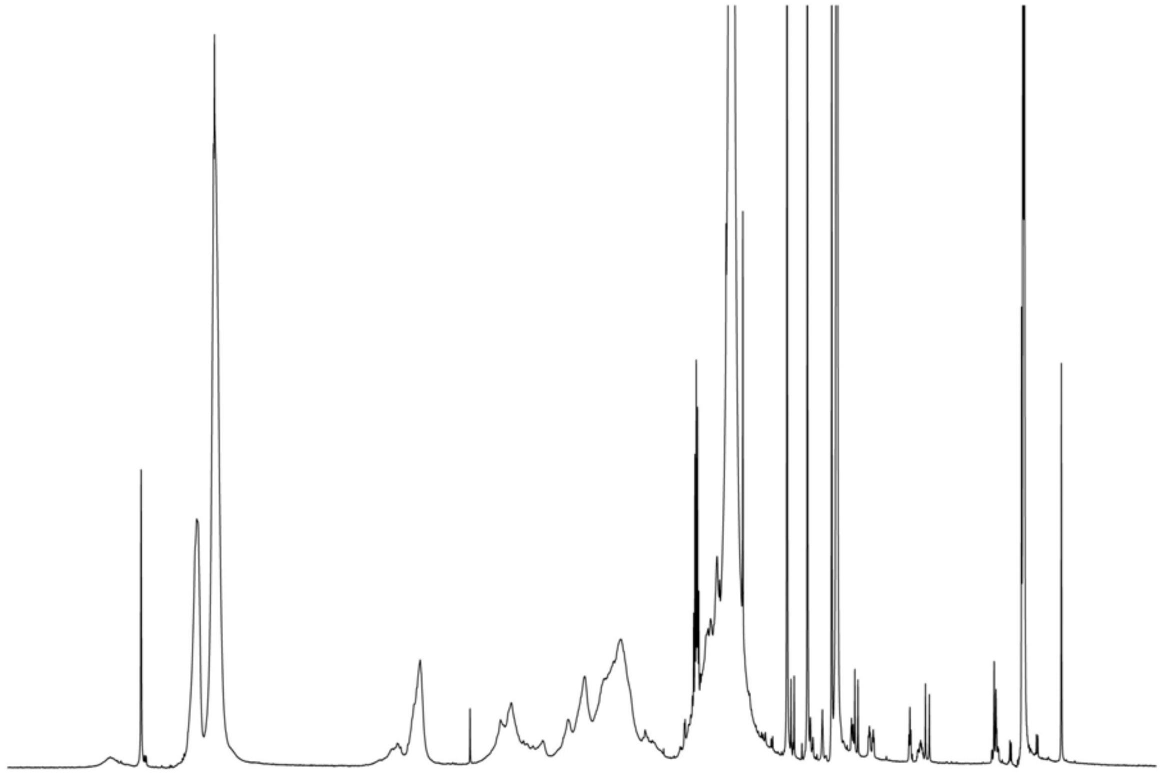

Figure S12: ^{1}H NMR spectrum (600 MHz, DMSO-d_6, 298 K) of **7-ii**

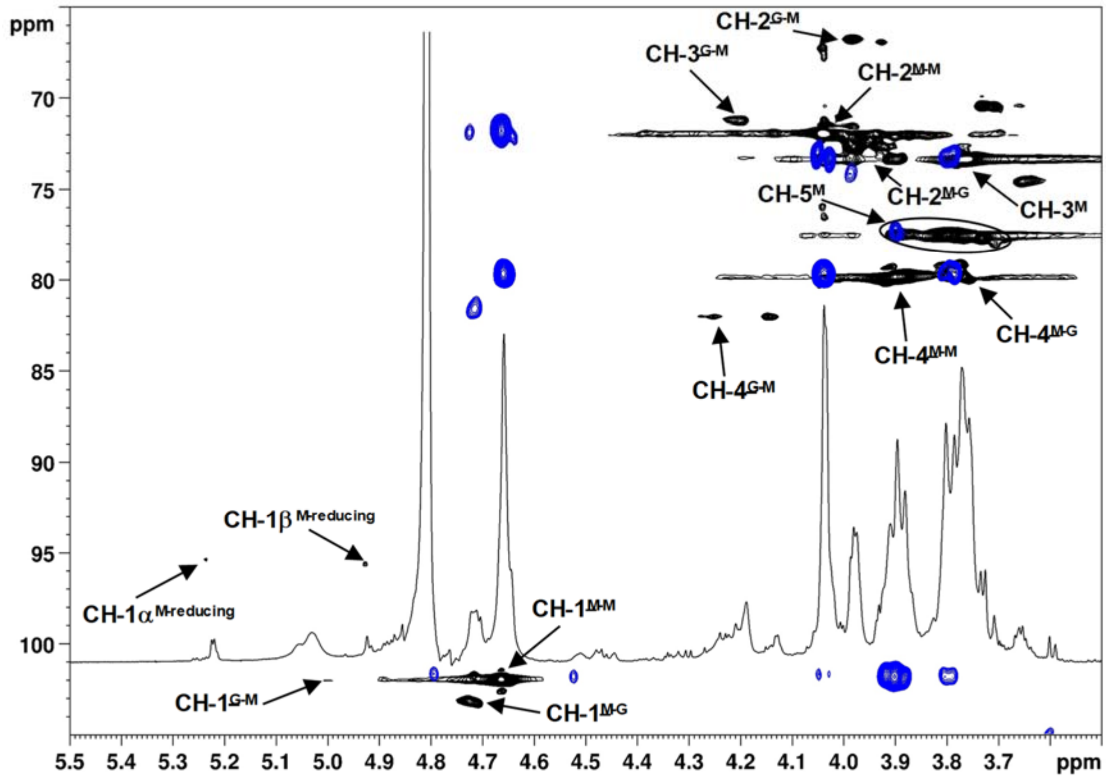

Figure S13: ^{1}H, ^{1}H,^{13}C-HSQC (black) and ^{1}H,^{13}C-HSQC (blue) NMR spectra (600 MHz, 298K, D_2O) of M-rich alginic acid (underlined letter M or G refers to the residue associated to the signal in the diad)

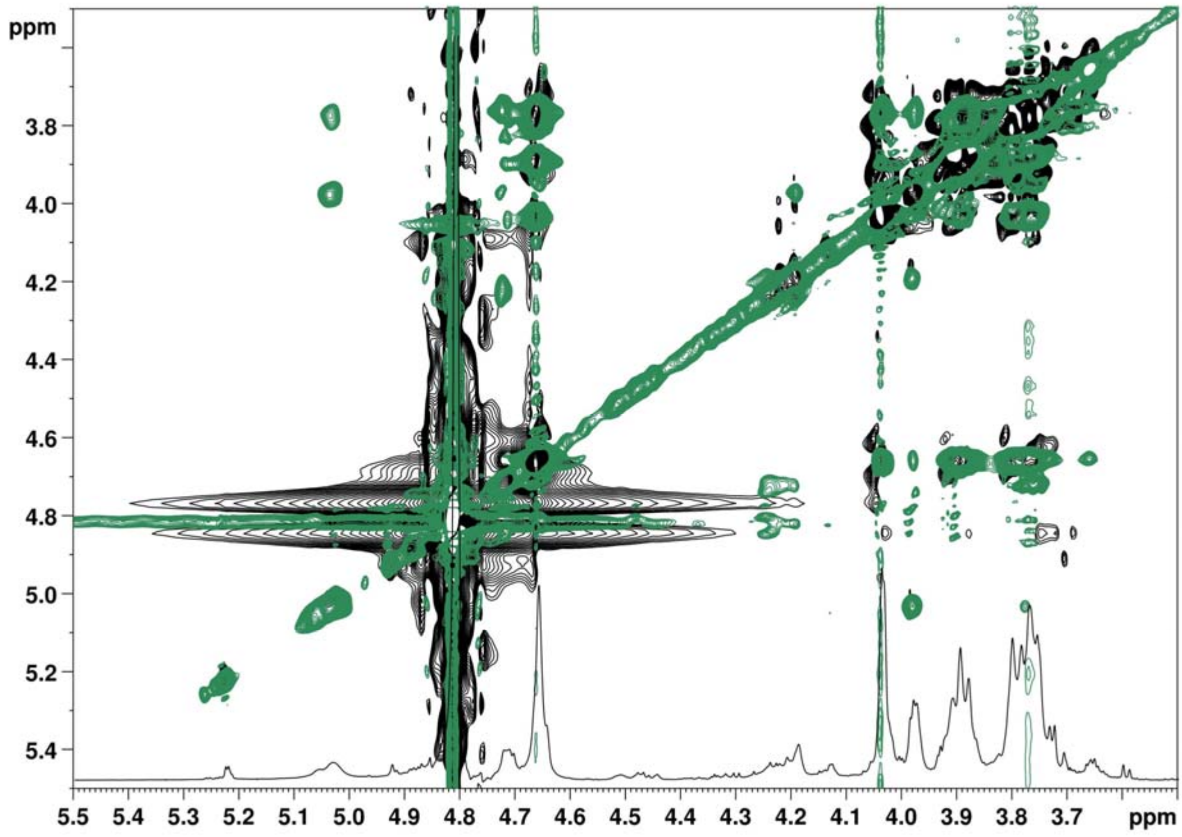

Figure S14: ^{1}H, COSY (black) and NOESY (green) NMR spectra (600 MHz, 298K, D_2O) of M-rich alginic acid

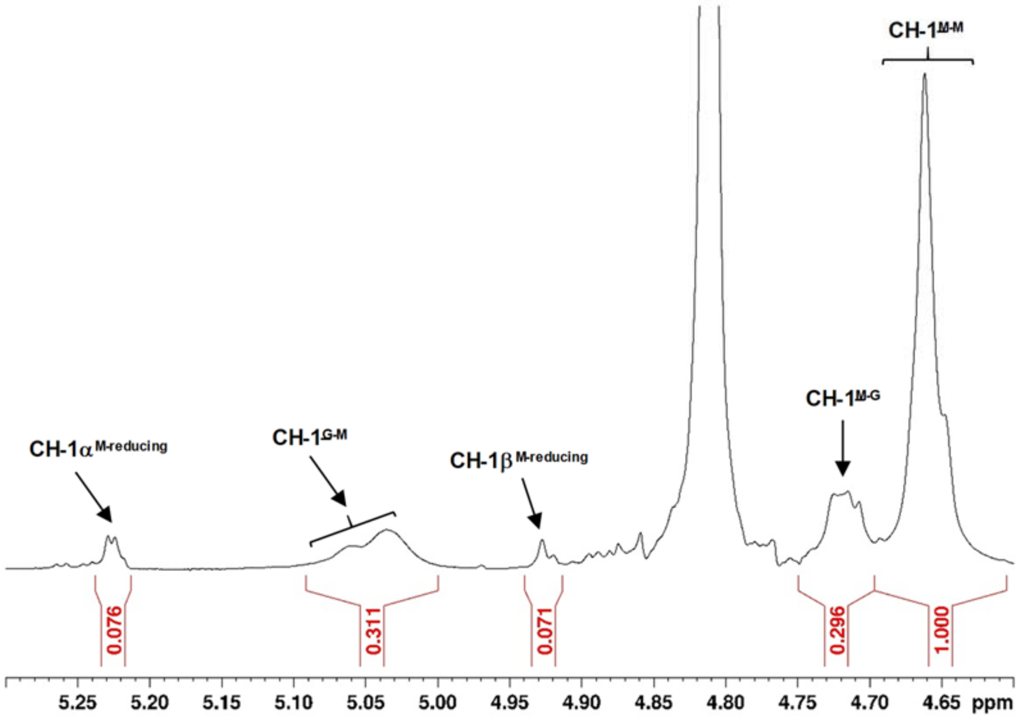

Figure S15: Zoomed ^{1}H NMR spectrum (600 MHz, 298K, D_2O) of M-rich alginic acid (underlined letter M or G refers to the residue associated to the signal in the diad)

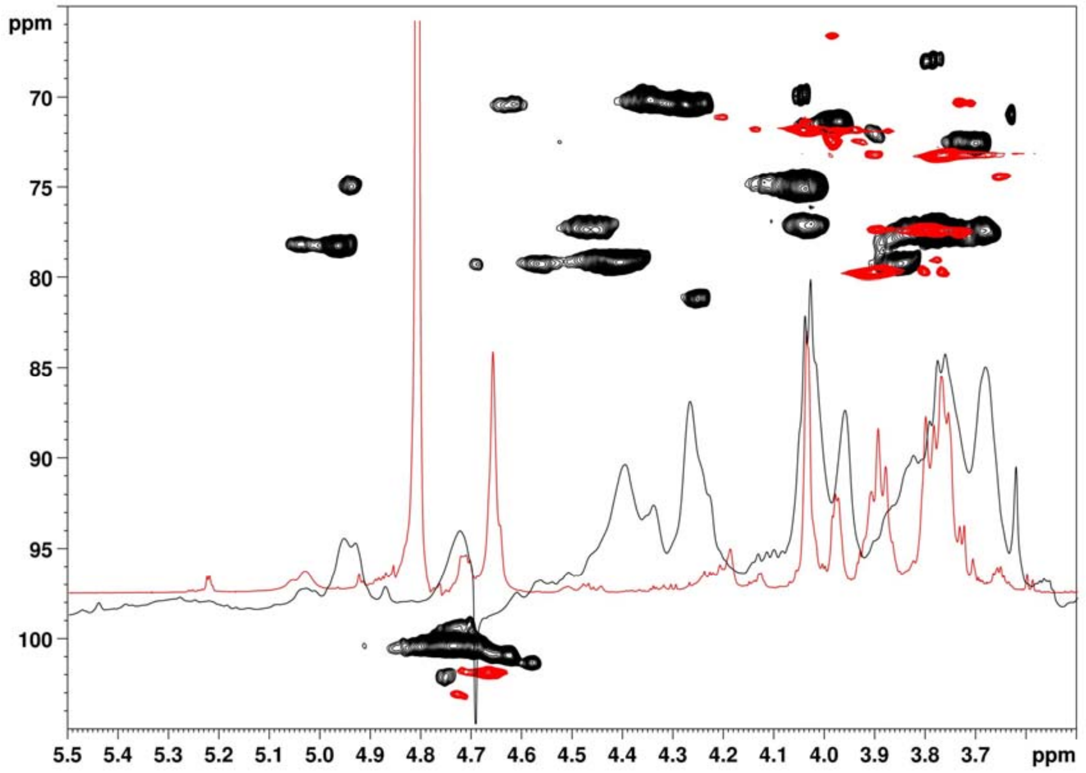

Figure S16: ^{1}H-NMR and ^{1}H,^{13}C-HSQC NMR spectra (600 MHz, 298K, D_2O) of **AS-1** (black) and M-rich alginic acid (red)

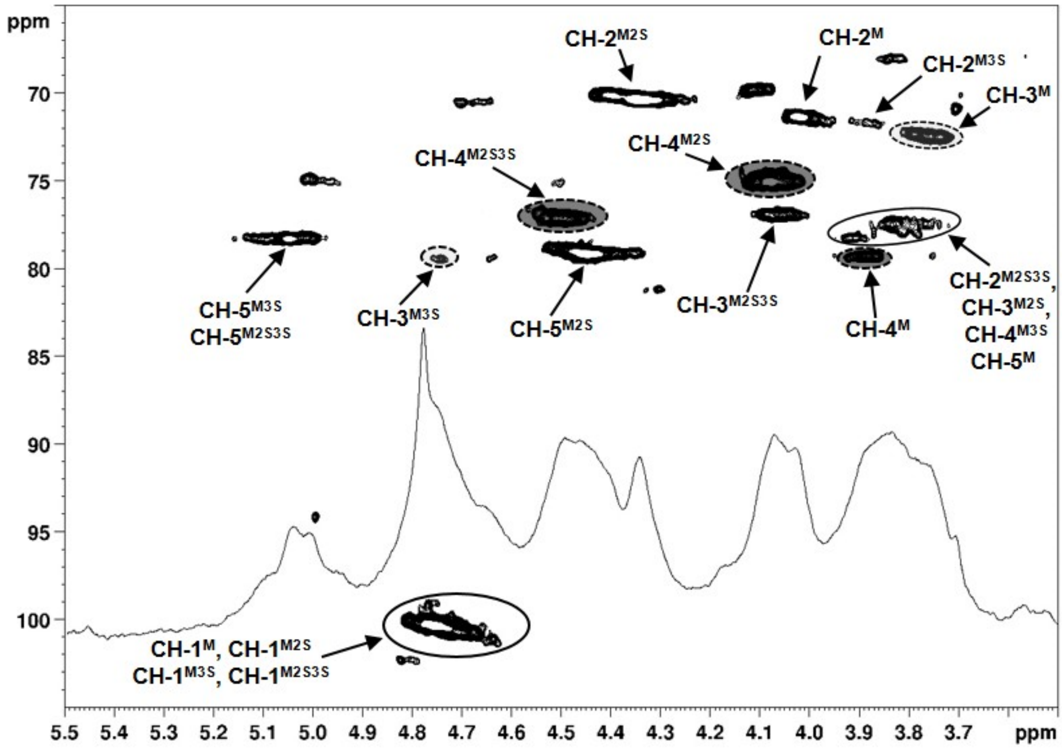

Figure S17: ^{1}H and ^{1}H,^{13}C-HSQC NMR spectra (600 MHz, 298K, D_2O) of **AS-2**

(densities enclosed in dotted lines and with the same filling were subjected to relative integration for DS estimation)

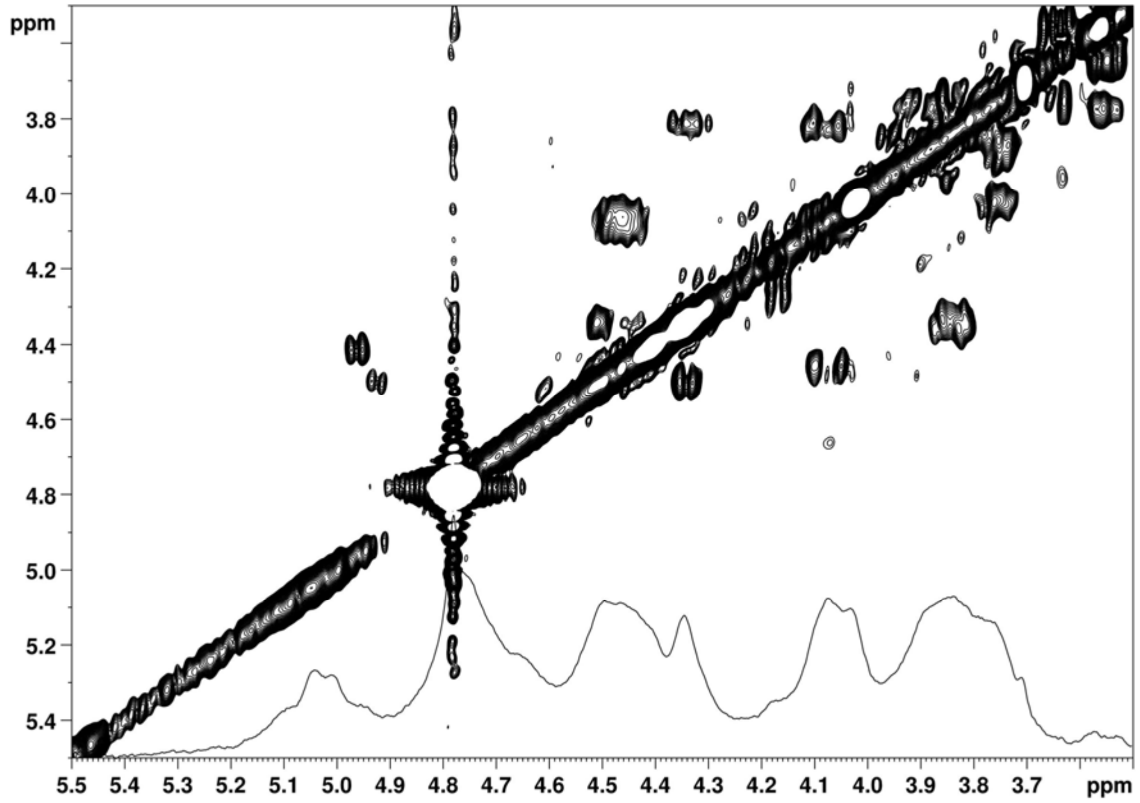

Figure S18: ^{1}H and COSY NMR spectra (600 MHz, 298K, D_2O) of **AS-2**

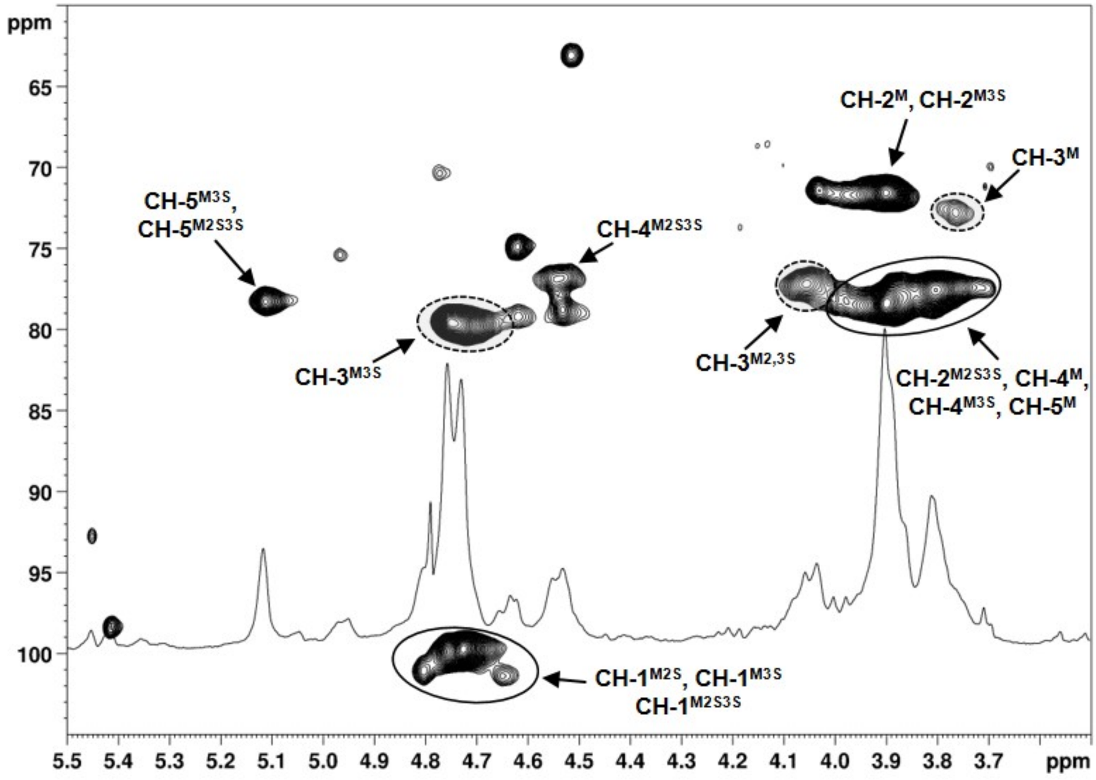

Figure S19: ^{1}H and $^{1}H,^{13}C$-HSQC NMR spectra (400 MHz, 298K, D_2O) of **AS-3**

(densities enclosed in dotted lines and with the same filling were subjected to relative integration for DS estimation)

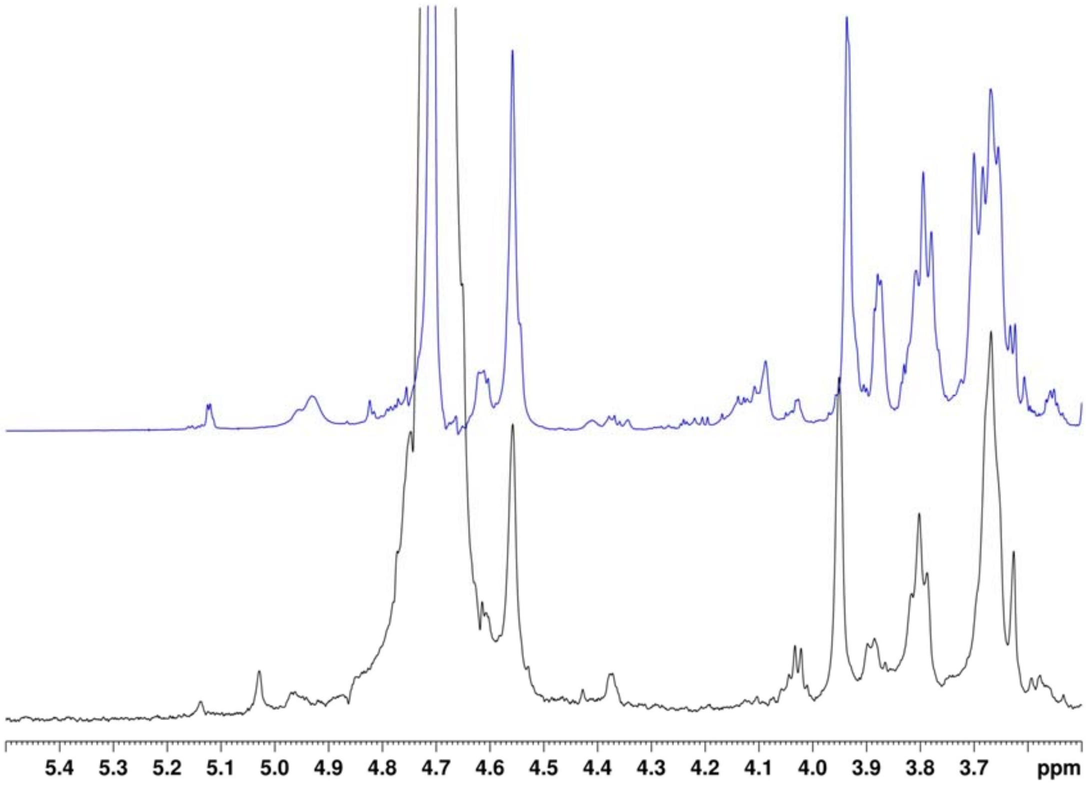

Figure S20: ^{1}H-NMR spectra (600 MHz, 298K, D_2O) of **AS-4** (black) and M-rich alginic acid (blue)

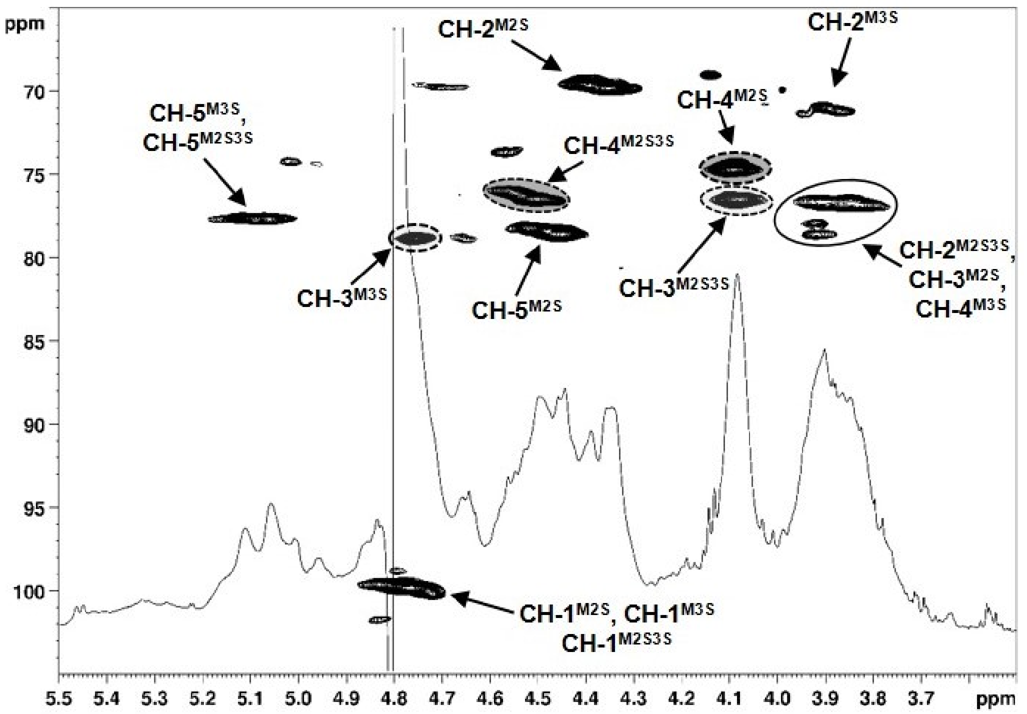

Figure S21: ^{1}H and ^{1}H,^{13}C-HSQC NMR spectra (400 MHz, 298K, D_2O) of **AS-5**

(densities enclosed in dotted lines and with the same filling were subjected to relative integration for DS estimation)

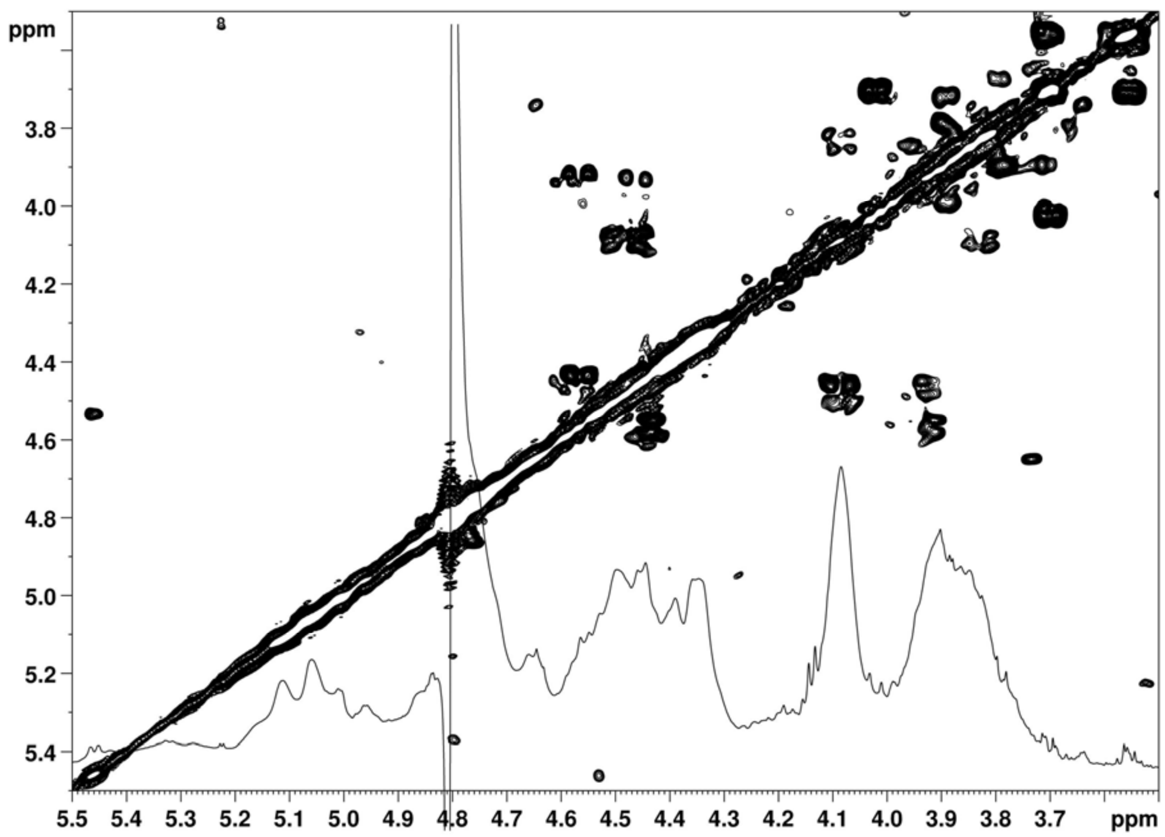

Figure S22: ^{1}H and COSY NMR spectra (400 MHz, 298K, D_2O) of **AS-5**

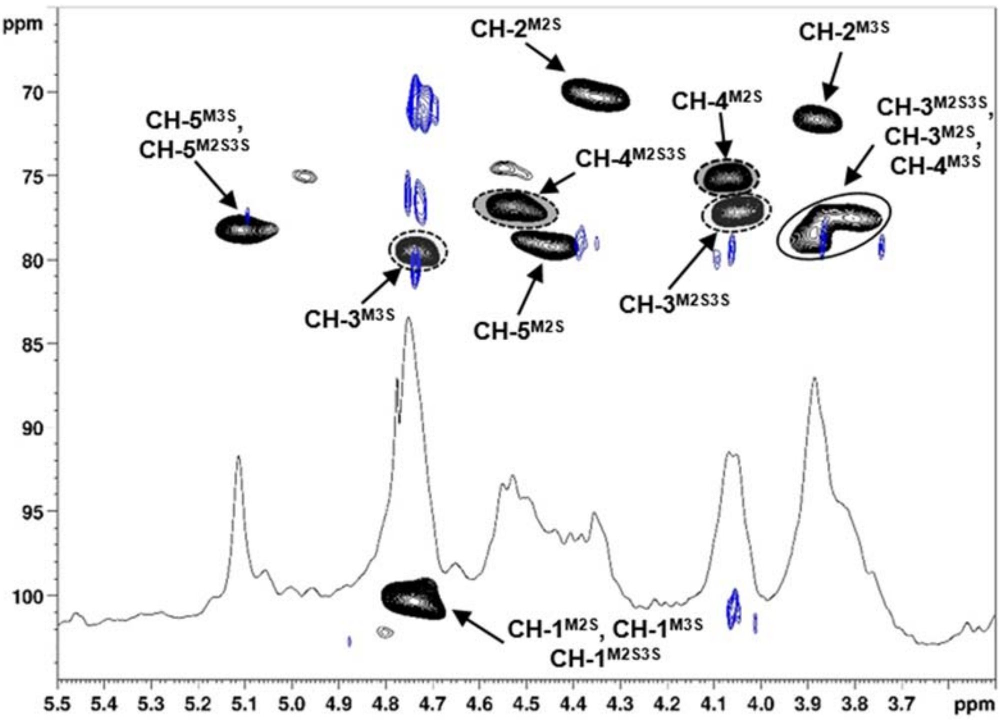

Figure S23: ^{1}H, ^{1}H,^{13}C-HSQC (black) and ^{1}H,^{13}C-HMBC (blue) 2D-NMR spectra (400 MHz, 298K, D_2O) of **AS-6**

(densities enclosed in dotted lines with the same filling were subjected to relative integration for DS estimation)

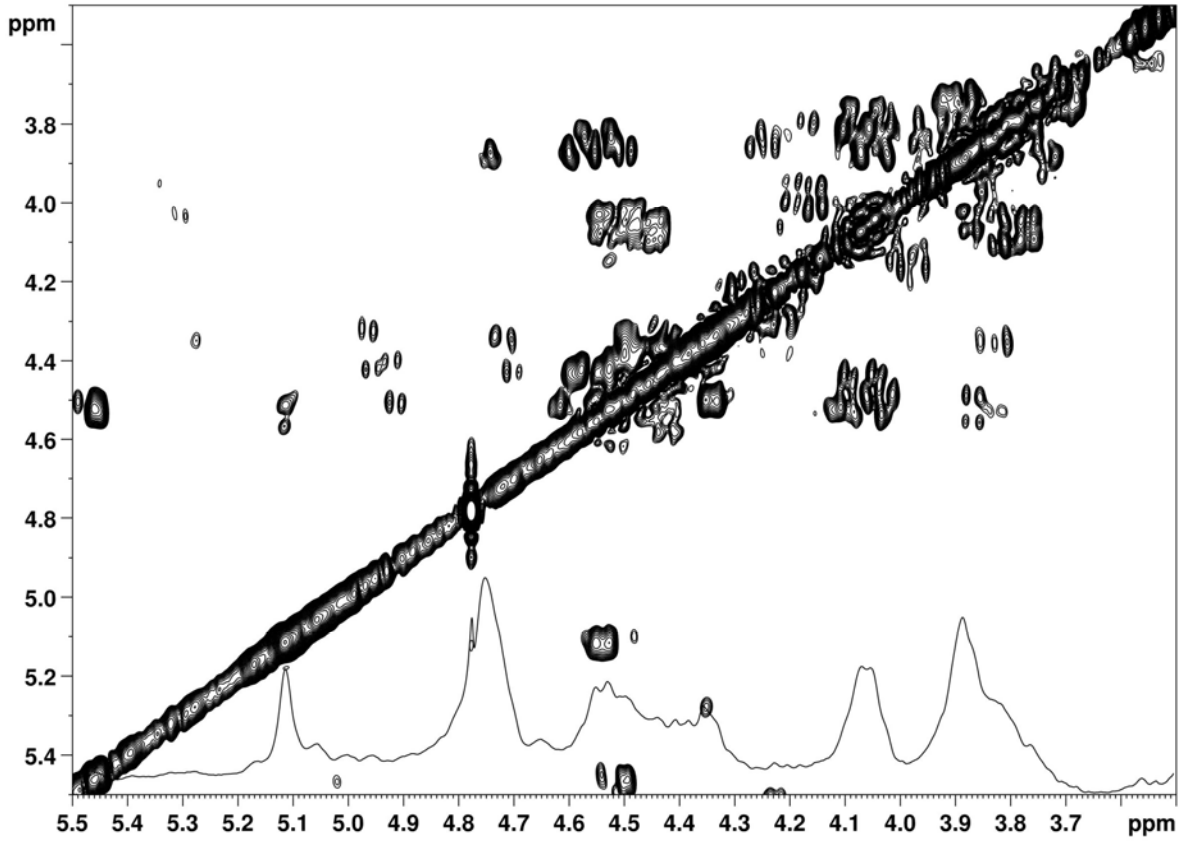

Figure S24: ^{1}H and COSY NMR spectra (400 MHz, 298K, D_2O) of **AS-6**

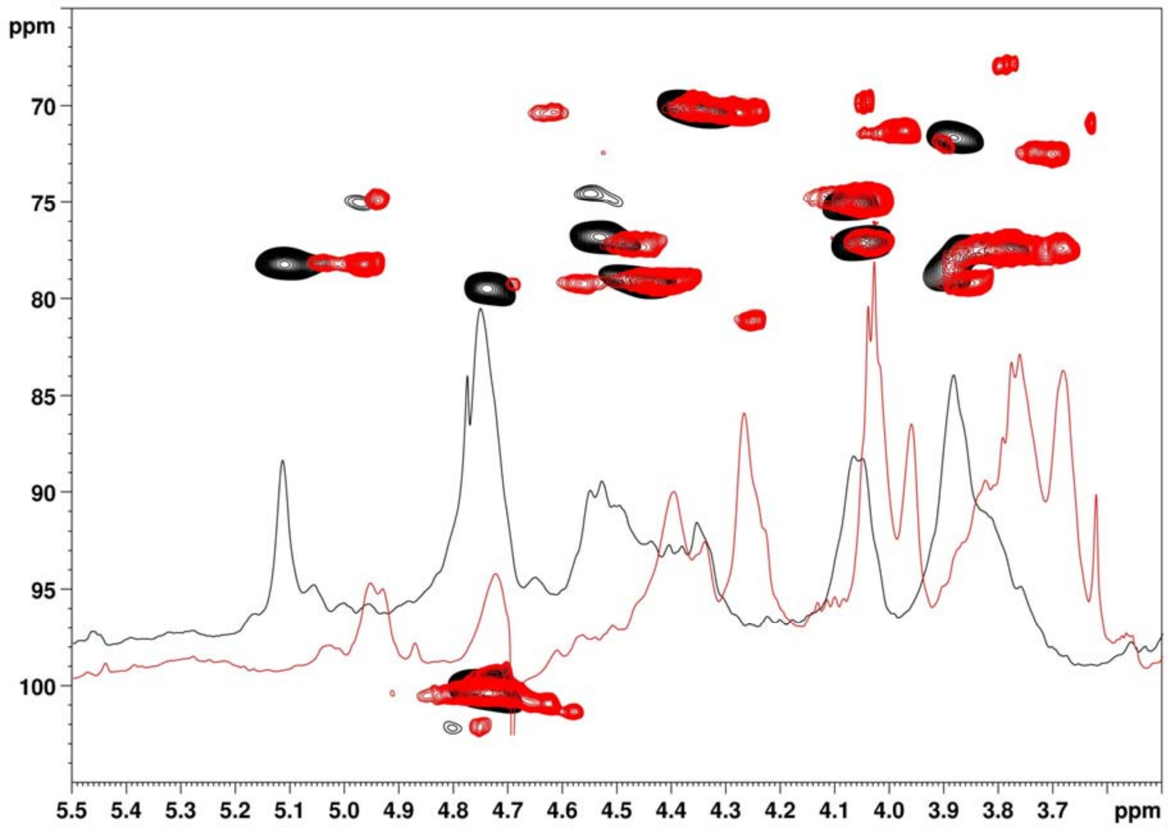

Figure S25: ^{1}H-NMR and ^{1}H,^{13}C-HSQC NMR spectra (400 MHz, 298K, D_2O) of **AS-6** (black) and **AS-1** (red)

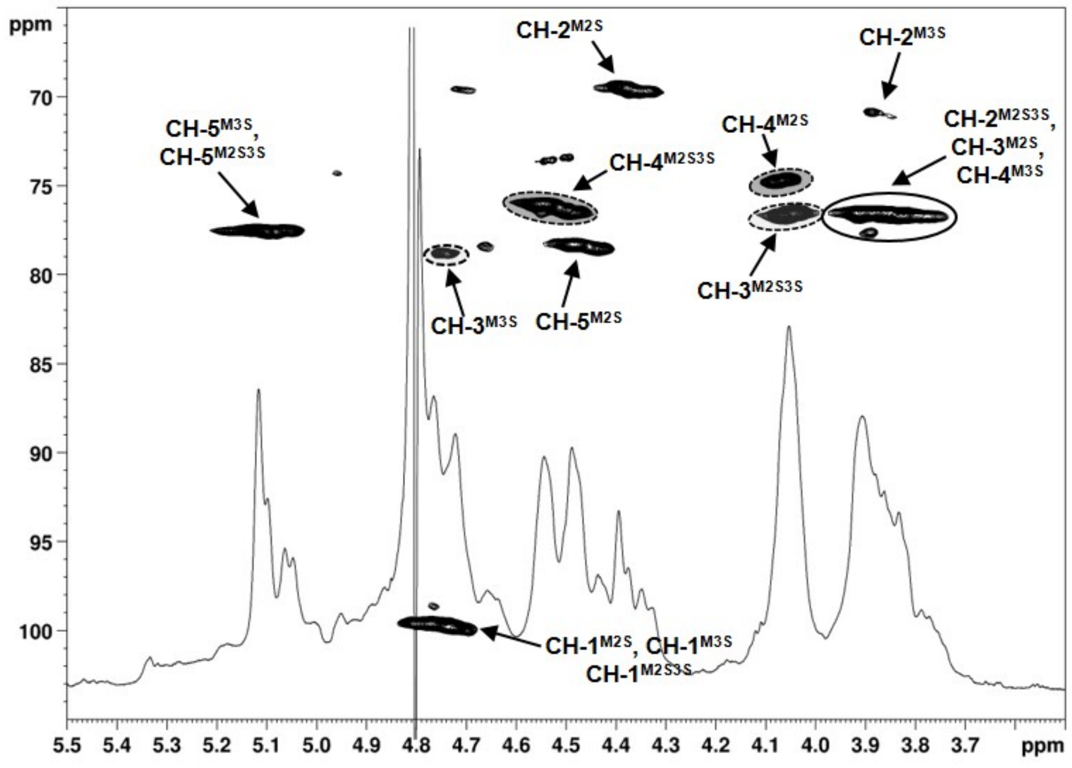

Figure S26: ^{1}H and ^{1}H,^{13}C-HSQC NMR spectra (400 MHz, 298K, D_2O) of **AS-7**
(densities enclosed in dotted lines and with the same filling were subjected to relative integration for DS estimation)

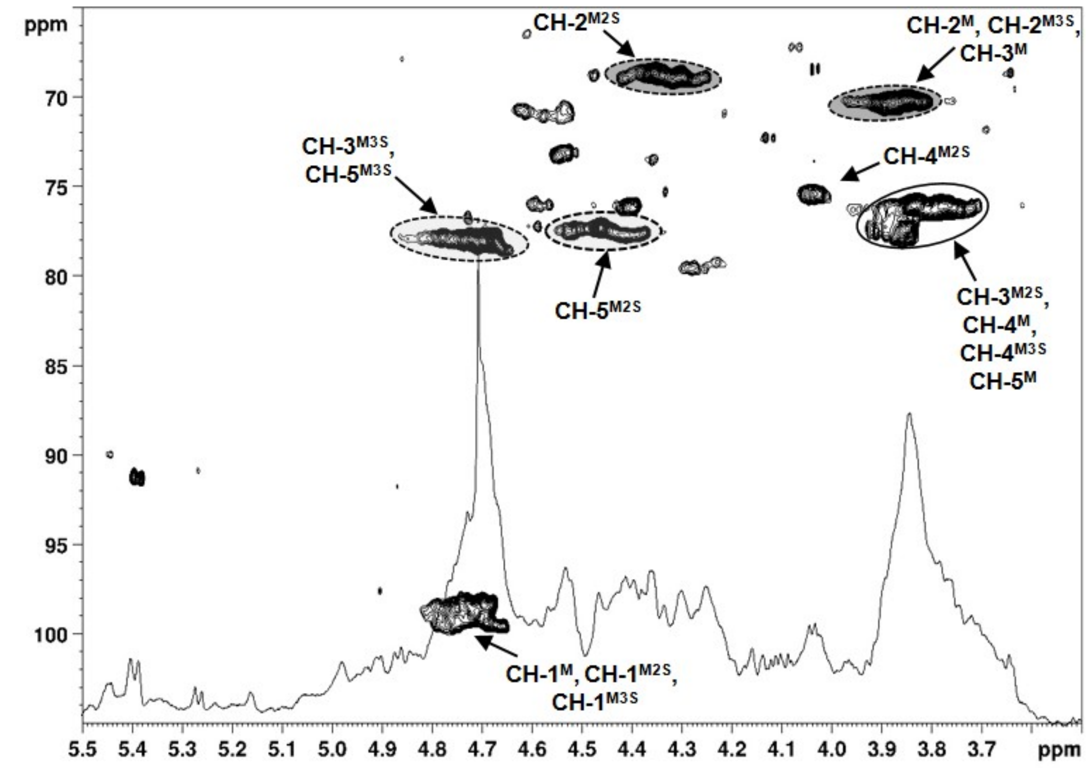

Figure S27: ^{1}H and ^{1}H,^{13}C-HSQC NMR spectra (400 MHz, 298K, D_2O) of **AS-8**

(densities enclosed in dotted lines and with the same filling were subjected to relative integration for DS estimation)

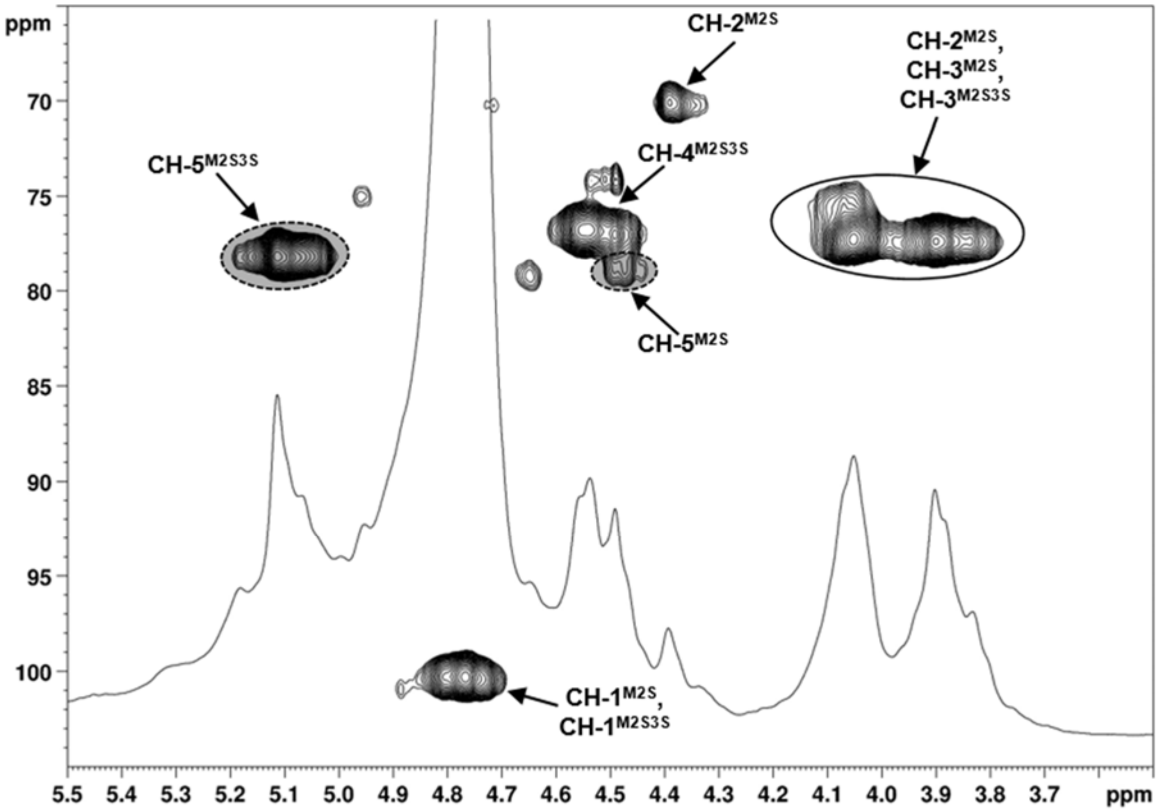

Figure S28: ^{1}H and ^{1}H,^{13}C-HSQC NMR spectra (400 MHz, 298K, D_2O) of **AS-9**

(densities enclosed in dotted lines were subjected to relative integration for DS estimation)

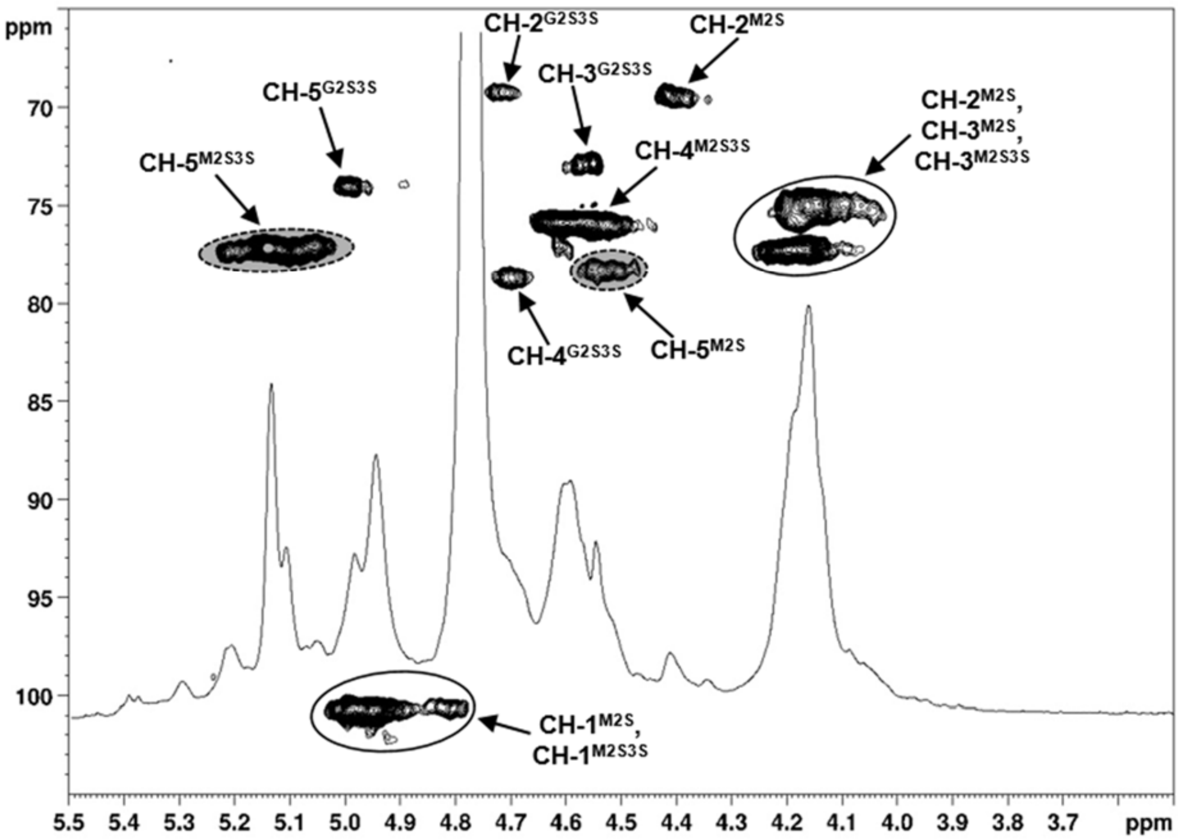

Figure S29: ^{1}H and $^{1}H,^{13}C$-HSQC NMR spectra (400 MHz, 298K, D_2O) of **AS-10**
(densities enclosed in dotted lines were subjected to relative integration for DS estimation)
